# FBXW2 inhibits prostate cancer proliferation and metastasis via promoting EGFR ubiquitylation and degradation

**DOI:** 10.1007/s00018-022-04320-3

**Published:** 2022-05-02

**Authors:** Tao Zhou, Tingting Chen, Bin Lai, Wenfeng Zhang, Xi Luo, Ding Xia, Weihua Fu, Jie Xu

**Affiliations:** 1 Department of Urology, The Second Affiliated Hospital, Third Military Medical University (Army Medical University), Chongqing, People’s Republic of China; 2 Department of Gastrointestinal Surgery, The Second Affiliated Hospital of Nanchang University, Nanchang, People’s Republic of China; 3 Department of Infectious Disease, The First Affiliated Hospital, Nanchang University, Nanchang, People’s Republic of China; 4 Department of Oncology, The First Affiliated Hospital, Third Military Medical University (Army Medical University), Chongqing, People’s Republic of China; 5 Department of Urology, Tongji Hospital, Tongji Medical College, Huazhong University of Science and Technology, Wuhan, People’s Republic of China

**Keywords:** FBXW2, Prostate cancer, EGFR, Ubiquitylation and degradation, Metastasis

## Abstract

FBXW2 is a poorly characterized F-box protein, as a tumor suppressor that inhibits growth and metastasis of lung cancer by promoting ubiquitylation and degradation of oncogenic proteins, including SKP2 and β-catenin. However, what the biological functions of FBXW2 in prostate cancer cells and whether FBXW2 targets other substrates to involve in progression of prostate cancer is still unclear. Here, we reported that overexpression of FBXW2 attenuated proliferation and metastasis of PCa models both in vitro and in vivo, while FBXW2 depletion exhibited the opposite effects. Intriguingly, FBXW2 was an E3 ligase for EGFR in prostate cancer. EGFR protein level and its half-life were extended by FBXW2 depletion, while EGFR protein level was decreased, and its half-life was shortened upon overexpression of FBXW2, but not its dominant-negative mutant. Importantly, FBXW2 bond to EGFR via its consensus degron motif (TSNNST), and ubiquitylated and degraded EGFR, resulting in repression of EGF function. Thus, our data uncover a novel that FBXW2 as a tumor suppressor of prostate cancer, inhibits EGFR downstream by promoting EGFR ubiquitination and degradation, resulting in repression of cell proliferation and metastasis.

**Supplementary Information:**

The online version contains supplementary material available at 10.1007/s00018-022-04320-3.

## Introduction

Prostate cancer (PCa) is the most common non-skin cancer and the second leading cause of cancer death in men [1]. Prostate specific antigen (PSA) has greatly improved the early diagnosis of PCa, and the 5-years survival rate of early PCa has reached nearly 100% [2]. However, the 5-years survival rate is low (28%) in progressive PCa, manifesting as bone metastasis and accompanying hypercalcemia, intractable pain, fracture and nerve compression [3]. Thus, metastasis is the leading cause of death in the majority of PCa patients [4].

Dysregulation of EGFR enhances bone metastases in many solid cancers [5], including PCa [6], but the molecular mechanisms by EGFR which supports the disease progression and metastasis is not fully understood. It is known that EGF binds to EGFR and induces the formation of EGFR dimer, which in turn promotes its autophosphorylation and internalization, leading to the activation of multiple oncogenic intracellular signaling pathways [7]. Therefore, EGFR has been used as an effective therapeutic target for tyrosine kinase inhibitors in pancreatic and lung cancers [8, 9]. On the other hand, by reducing tumor suppressor miR-1 and activating oncogene TWIST1, EGFR promoted progression and bone metastasis of PCa [5], as well as EGFR expression in bone metastasis [10]. Casitas Blineage lymphoma (c-Cbl) has been reported as an E3 ligase of EGFR by promoting EGFR ubiquitylation and endocytosis-based degradation upon EGF stimulation [11, 12]. However, a recent study indicated that down-regulation of c-Cbl had no effect on EGFR expression [13], which suggests that the degradation mechanisms of EGFR merit further investigation.

To pursue additional involving genes, we recently reported F-box and WD-repeat domain-containing 2 (FBXW2), a poorly characterized F-box protein, whose function is a substrate recognition receptor in the SKP1-Cullin1-F-box protein (SCF) ubiquitin ligase complexes [14]. The SCF ubiquitin ligases, also known as Cullin-RING ligase 1 (CRL1), consist of adapter protein SKP1, scaffold protein Cullin-1, Ring box protein-1 (RBX1)/ROC1, and an F-box protein. While Cullin and RING protein are required for ligase activity, the F-box protein determines the substrate specificity and emerges as an important player in tumorigenesis [15, 16]. Glial cell missing homolog 1 (GCM1) is an important transcription factor regulating placental cell fusion, recently, has been demonstrated that GCM1 is ubiquitinated and degraded by SCF-FBXW2 E3 ligase complex [17]. On the other hand, FBXW2 also has been confirmed to inhibit the tumor growth and metastasis of lung cancer by promoting ubiquitylation and degradation of β-catenin and S phase kinase-associated protein 2 (SKP2) [15, 18]. However, what the biological functions of FBXW2 in PCa cells and whether FBXW2 targets other substrates to regulate the proliferation and metastasis of PCa cells is totally unknown.

Here, we reported that FBXW2 was an E3 ligase for EGFR. FBXW2 binds to EGFR upon its consensus degron motif (TSNNST), and promoted EGFR ubiquitylation and degradation. Overexpression of FBXW2 attenuated growth and metastasis in both in vitro and in vivo PCa models, whereas depletion of FBXW2 had opposite effects. Thus, FBXW2 inhibited EGFR downstream by targeting for EGFR ubiquitination and degradation, resulting in repression of PCa cell proliferation and metastasis.

## Materials and methods

### Cell culture

All cell lines used in this study were obtained from the Zhong Qiao Xin Zhou Biotechnology Co., Ltd. (Shanghai, China) and were authenticated by short tandem repeat profiling and monitored Mycoplasma contamination. 22RV1, LNCaP, PC3, and DU145 PCa cell lines were routinely cultured in RPMI-1640 medium (#21870076, Gibco). RWPE cell line was maintained in KSFM medium (#10744019, Gibco). 293 T cells were grown in DMEM medium (#11965092, Gibco). All mediums were supplemented with 10% fetal bovine serum (FBS) and 1% penicillin–streptomycin. All the medium was purchased from Gibco. All cell lines were incubated at 37 °C with 5% carbon dioxide.

### Cell counting Kit-8 (CCK-8) assay

The cells concentration was adjusted to around 2 × 10^3^ cells/well, and the cells were seeded into 96-well plates in 100 μL of culture medium, followed by incubation at 37 °C in an atmosphere with 5% carbon dioxide cultivation. At various time points, 10 μL of CCK-8 reagent (HY-K0301, MCE) at a 1:10 dilution with serum-free RPMI-1640 medium was added to each well, followed by a further 2 h incubation. The cell viability was determined using the CCK-8 assay, and the optical density (OD) was measured at 450 nm. Each experiment was conducted in triplicates.

### Plasmids, siRNA, and transfection

The pEX-1 plasmid vector used in this study carried an enhanced green fluorescence protein (eGFP), and HA-tag or FLAG-tag added in 5' upstream of the target gene as needed. The construction and quality inspection of the plasmid was completed by Sangon Biotech (Shanghai, China). The targeting plasmids were delivered into PC3 and DU145 cells using the Lipofectamine 2000 transfection reagent (#11668019, Invitrogen, USA) according to the manufacturer’s instructions. G418 (PC3 with 100 μg/mL, DU145 with 120 μg/mL, Gibco, USA) was used to screen individual clone expressing FBXW2 or vector control. siRNA designed to target FBXW2 were purchased from Sangon Biotech (Shanghai, China), including siRNA-FBXW2-#970 (5'-CCUCUUAAGUGCAGACAAATT-3'), siRNA-FBXW2-#1014 (5'-GGAGA GAAAUCAACUGUAATT-3') and siRNA-FBXW2-#1174 (5'-CAUCAAGACUC CUGAGAUATT-3'). For transient transfection, control and FBXW2 siRNA (#970, #1014 and #1174) were mixed with Lipofectamine 2000 and then added to cell culture medium in 22RV1 and LNCaP cells according to the manufacturer's instructions. Finally, we used immunoblotting (IB) to evaluate the transfection efficiency.

### RNA isolation, reverse-transcription, and qPCR

Total RNA was isolated by Trizol–Chloroform method and then transcribed into cDNA using reverse-transcription kit (Takara, Japan) with an oligo (dT) 20 bp primer. RT-qPCR was performed using the SYBR green reagent (Takara, Japan) on Real-Time PCR System (Thermo Fisher, USA). The 2^−ΔΔCt^ method was used to quantify the data, and the housekeeping GAPDH was used as loading control in the analysis. The sequences of primer sets were 5'-GGACATGCCTGAACACACTC-3' and 5'-CCAGGACTGTGCAAGAGAGA-3' for FBXW2; 5'-ACCCAGAAGACTG TGGATGG-3' and 5'-TCAGCTCAGGGATGACCTTG-3' for GAPDH. Each experiment was conducted in triplicates.

### Wound-healing assay

Culture-Insert (Ibidi, Germany) was placed in the 12-well plate and made the sticky side stick to the bottom of the well. The cells were counted, diluted to 70–100 × 10^4^ cells/mL, and 70μL suspension was added to the slots on both sides of the Culture-Insert. Vertically removed the Culture-Insert 24 h later, and a straight scratch was formed between the cells in the slots. Plate was washed by PBS and the cells were continually incubated in serum-free medium. Photographs of the scratch were taken at various time points by inverted microscope (Olympus, Japan). The mean value of each gap width was measured for statistical analysis. Each experiment was conducted in triplicates.

### Invasion assay

Matrigel (Corning, USA) was diluted with five times serum-free cell medium, and 50 μL of which was added to the bottom of each transwell chamber and incubated for 4 h. The Matrigel-coated chambers were washed with serum-free medium for later use. The chambers were placed in a 24-well plate, in each of them 4 × 10^4^ cells in 200 μl medium containing 2% FBS free medium were plated, together with medium containing 10% FBS at the bottom of the well. Cells were incubated for 24–48 h, and then fixed with 4% paraformaldehyde for 20 min. After washing with PBS for three times, cells were stained with 0.1% crystal violet (Beyotime, China) for 20 min, and then washed with PBS. Cells on the upper surface of the chamber were removed by cotton swab. The positively stained cells were photographed under the microscope, and multiple fields were taken for cell count and statistics. Each experiment was conducted in triplicates.

### Apoptosis and cell cycle assays

Cells were synchronized at G0/G1 by serum starvation for 24 h and then released by serum addition. For cell apoptosis detection, cells both in adherent and supernatant were collected and were stained in dark with 5 μL Annexin v-fitc (Gibco, USA) for 10 min, and then labeled with 10 μL FxCycleTM PI/RNase for 5 min before flow cytometry. For cell cycle detection, cells were collected when cell density reached 70–90%, and fixed in 70% ice-cold ethanol overnight, labeled with 500 μL FxCycleTM PI/RNase (Gibco, USA) for 15–30 min in dark at room temperature, and analyzed directly on an LSR II flowcytometer (BD Biosciences, USA). Each experiment was conducted in triplicates.

### Immunoblotting (IB) and immunoprecipitation (IP)

For direct IB analysis, cells were lysed in RIPA buffer with protease inhibitors and phosphatase inhibitors (Roche Life Science, Switzerland). The following primary antibodies were used: rabbit-FBXW2 (ab83467, Abcam; 1:1000 overnight, 4 °C); rabbit-FBXW2 (11499-1-AP, proteintech; 1:500 overnight, 4 °C); rabbit-EGFR (#4267, CST; 1:1000 overnight, 4 °C); rabbit-caspase 3 (19677–1-AP, proteintech, 1:1000 overnight, 4 °C); rat-HA (#11867423001, Roche Life Science, 1:2000 overnight, 4 °C); mouse-FLAG (#F1804, Sigma, 1:2000 overnight, 4 °C); mouse-His-Tag (66005-1-Ig, proteintech, 1:1000 overnight, 4 °C); rabbit-AR (#5153, CST, 1:1000 overnight, 4 °C); rabbit-p-AKT (#4060S, CST, 1:1000 overnight, 4 °C); rabbit-AKT (#4691S, CST, 1:1000 overnight, 4 °C); rabbit-Cyclin B1 (#12231S, CST, 1:1000 overnight, 4 °C); rabbit-Cyclin D1 (#2978, CST, 1:1000 overnight, 4 °C); rabbit-Cyclin E1 (11554-1-AP, proteintech, 1:1000 overnight, 4 °C); rabbit-p-STAT3 (#9145, CST, 1:1,000 overnight, 4 °C); mouse-STAT3 (ab119352, Abcam, 1:1,000 overnight, 4 °C); rabbit-BAX (50599-2-Ig, proteintech, 1:1000 overnight, 4 °C); rabbit-BCL2 (12789-1-AP, proteintech, 1:1000 overnight, 4 °C); rabbit-PARP1 (13371-1-AP, proteintech, 1:1000 overnight, 4 °C); rabbit-p27 (25614-1-AP, proteintech, 1:1000 overnight, 4 °C); rabbit-p21 (103551-1-AP, proteintech, 1:1000 overnight, 4 °C); mouse-GAPDH (60004-1-Ig, proteintech, 1:2000 overnight, 4 °C); mouse-β-Actin (60008-1-Ig, proteintech, 1:5000 overnight, 4 °C); mouse-α-Tubulin (66031-1-Ig, proteintech, 1:5000 overnight, 4 °C). For immune-precipitation, cells were treated by MG132 (10 μg/mL, Beyotime, China) for 4 h before lysed in IP-RIPA buffer with protease inhibitors, and Protein A/G plus Agarose (Santa Cruz, USA) were used to pull down the proteins. To immunoprecipitate endogenous proteins, whole cell extracts were pre-cleared with normal IgG-AC (Santa Cruz, USA) followed by overnight incubation at 4 °C with antibody against HA-Tag. To immunoprecipitate exogenously expressed FLAG-Tag proteins, the pre-cleared cell lysates were incubated with HA-Tag antibody in a rotating incubator overnight at 4 °C. The Protein A/G plus agarose were washed with NETN-100 and the co-precipitated proteins were assessed by IB. Each experiment was conducted in triplicates.

### Half-life analysis

After gene manipulation, 20 μg/mL cycloheximide (CHX, MCE, China) was added to the cell medium for inhibiting new protein synthesis. At the indicated time points, cells were harvested, lysed, and subjected to IB analysis. Each experiment was conducted in triplicates.

#### The in vivo ubiquitylation assay

All ubiquitin mutants and His–Ub plasmids were obtained from Dr. Yi Sun from University of Zhejiang. Briefly, PC3 cells were co-transfected with HA-FBXW2 and His-Ub to detect ubiquitination of endogenous EGFR. To detect ubiquitination of exogenous EGFR, 293 T cells were co-transfected with HA-FBXW2, His-Ub, and FLAG-EGFR-WT, FLAG-EGFR-MU1 or FLAG-EGFR-MU2. Mock vector was used as a control for ubiquitination assay. Cells were lysed in buffer A (6 M guanidine–HCl, 0.1 M Na_2_HPO_4_, 0.007 M NaH_2_PO_4_, and 5 mM imidazole, 0.1% Triton X-100, 10 mM β-mercaptoethanol, pH 8.0) and sonicated. The lysates were incubated with nickel–nitrilotriacetic acid (Ni–NTA) beads (QIAGEN, Germany) at 4 °C overnight. The beads were washed, respectively, with buffer A and buffer B (8 M urea, 0.1 M Na_2_HPO_4_, 0.007 M NaH_2_PO_4_, and 5 mM imidazole, 0.1% Triton X-100, 10 mM β-mercaptoethanol, pH 8.0), and then three times with buffer C (8 M urea, 0.025 M Na_2_HPO_4_, 0.075 M NaH_2_PO_4_, and 5 mM imidazole, 0.1% Triton X-100, 10 mM β-mercaptoethanol, pH 6.3). The beads were boiled and the pull-down proteins were resolved with anti-HA or anti-FLAG antibody by subsequently IB assay. Each experiment was conducted in triplicates.

#### Animal experiments

All experimental procedures using mice were performed in accordance with protocols approved by Laboratory Animal Welfare and Ethics Committee of Third Military Medical University of China. For xenograft model, 1 × 10^6^ PC3 stable cells (Vector and HA-FBXW2) were mixed 1:1 with Matrigel in a total volume of 0.2 mL and were injected subcutaneously into both flanks of BALB/c athymic nude mice (nu/nu, male; 4–6 week old; 13–15 g). The size of tumors and the weight of the mice were measured twice a week. Mice were killed by carbon dioxide asphyxiation on day 30 after tumor cell injection, when some of the tumors reached the size limit set by the Institutional Animal Care and Use Committee. Tumors were weighed and fixed with 4% paraformaldehyde after resection.

For intratibial injection model, 2.0 × 10^6^ PC3 stable cells (Vector and HA-FBXW2) were re-suspended in 100 µL PBS and 10 µL of cell solution was slowly injected into NOD/SCID mice (male 4–6 week old; 13–15 g) with a 28.5-G needle into the tibia using a drilling motion. Osteolytic lesions were identified on radiographs as radiolucent lesions in the bone. Each bone metastasis was scored as follows [19]: 0, no metastasis; 1, bone lesion covering less than 1/4 of the bone width; 2, bone lesion involving 1/4 to 1/2 of the bone width; 3, bone lesion across 1/2 to 3/4 of the bone width; and 4, bone lesion more than 3/4 of the bone width. The bone metastasis score of each mouse was the sum of all bone injuries. Mice were sacrificed dependent on survival time. Two hind limbs were dissected and fixed with 4% paraformaldehyde, which were subsequently used for H&E staining.

#### Clinical specimens and tissue microarray

All nine prostate samples were collected from the urological specimen bank of the second affiliated hospital of the army medical university. The establishment of the specimen bank met the ethical requirements, and each sample had complete case information and pathological results. Three prostate hyperplasia samples were in the control group, three prostate cancer samples with Gleason score ≤ 7 without metastasis were in the PCa group, and three prostate cancer samples with Gleason score of > 7 and metastasis were in the M-PCa group. Tissue microarray was purchased from Servicebio (Wuhan, China), which provided chip information and completed immunohistochemical staining of the chips.

#### Immunohistochemistry (IHC)

IHC staining of Ki-67, EGFR, p-AKT, p-STAT3, p27, and p21 proteins in tumors of the nude mice was performed as described previously [18, 20]. The tissues were paraffin-embedded and sectioned after formalin-fixation. Tissue sections were subjected to IHC by incubation with anti-Ki-67 (#9449, CST, 1:1000), anti-EGFR (#4267, CST, 1:200), anti-p-AKT (#4060, CST, 1:200), anti-p-STAT3 (#9145, CST, 1:200), anti-p27 (25614-1-AP, Proteintech, 1:200), and anti-p21 (10355-1-AP, Proteintech,1:200) antibodies, respectively, after deparaffinizing and antigen repairing. The sections were then incubated sequentially with biotinylated secondary antibody and HRP (horseradish peroxidase)-conjugated streptavidin. Antigenic detection was performed using chromogenic substrate DAB (3,3-diaminobenzidine tetrachloromethane) and the sections were counter stained further by hematoxylin. The positively stained cells were photographed under the microscope, and multiple fields were taken for cell count and statistics.

#### Statistical methods and chart

All data were expressed as Mean ± SD, SPSS 16.0 software was used for statistical analysis, and GraphPad Prism 7.0 software was used to make statistical charts. Differences between two groups were analyzed using unpaired Student’s *t* test. Comparisons of multiple groups were analyzed using one-way ANOVA or two-way ANOVA, followed by Dunnett’s test or Sidak’s test for evaluating the significance of differences between groups. *P* values less than 0.05 were defined as statistically significant.

## Results

### FBXW2 is down-regulated in highly-metastatic PCa cells and tissues, and enhanced FBXW2 expression attenuates cancer growth and metastasis in vitro

**Fig. 1:**
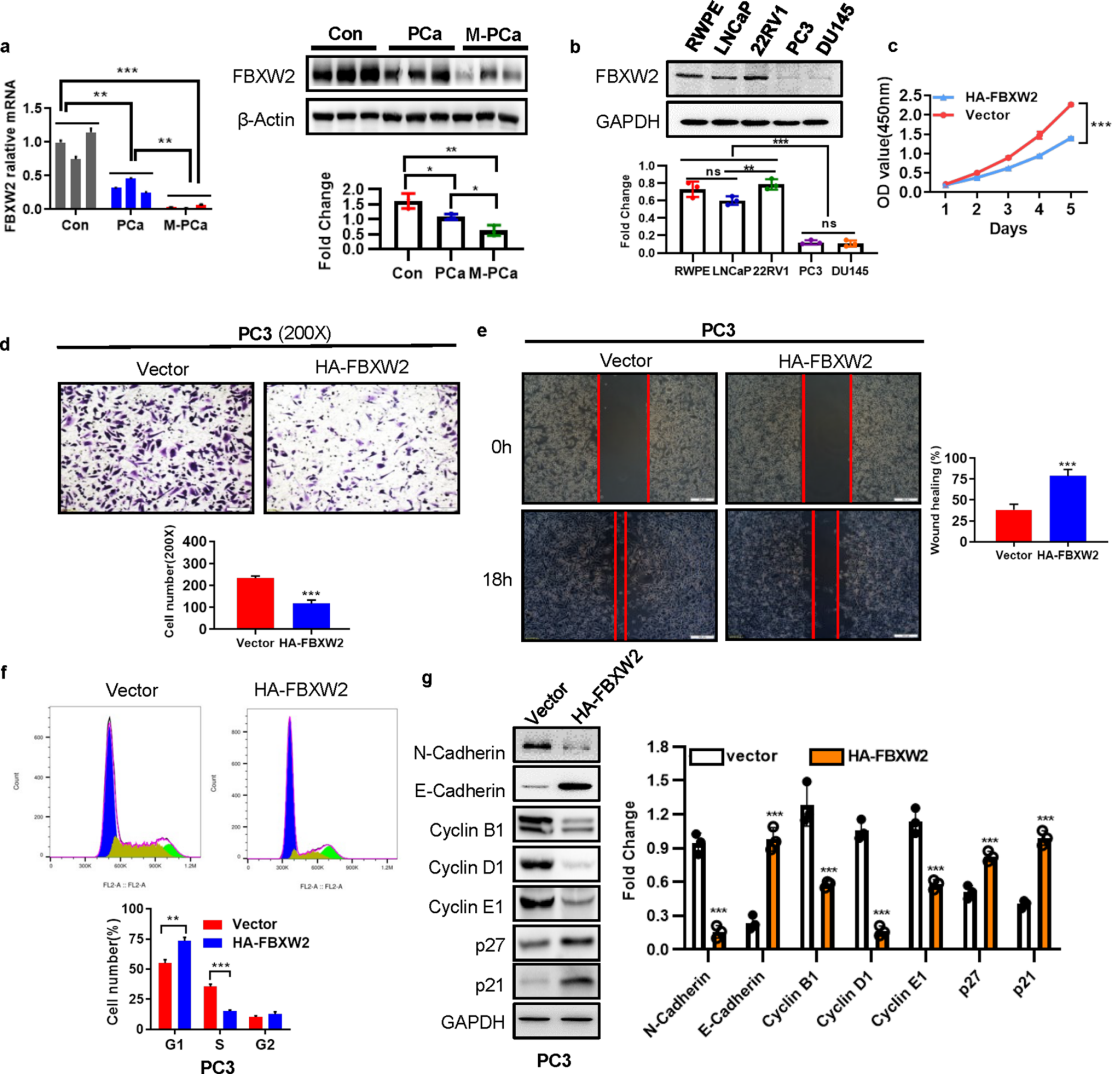
FBXW2 is down-regulated in highly-metastasis PCa cells and tissues, and enhanced FBXW2 expression inhibits cancer growth and metastasis. **a** Both mRNA and protein levels of FBXW2 were significantly decreased in highly-progressive PCa tissues. We collected 9 clinical samples and divided them into three groups: prostatic hyperplasia (Nor), Non-metastatic PCa (PCa, Gleason score ≤ 7) and metastatic prostate cancer (M-PCa, Gleason score > 7). Then, the mRNA and protein levels of FBXW2 in clinical samples were analyzed by qPCR analysis and immunoblotting (IB), respectively (*p < 0.05, **p < 0.01, ***p < 0.001; *n* = 3; one-way ANOVA). **b** FBXW2 protein levels in prostate cell lines were analyzed by IB analysis (ns, no significance, **p < 0.01, ***p < 0.001; n = 3; one-way ANOVA). **c**–**e** Overexpression of FBXW2 in PC3 cells inhibited cell proliferation (**c**), invasion (**d**), and migration (**e**). Scale bar:100 µm (**d**) and 200 µm (**e**). Cell viability was detected with Cell Counting Kit-8 (CCK-8) assay (***p < 0.001; *n* = 3; two-way ANOVA). The cell migration ability and invasion ability were determined using the wound-healing and transwell assays, respectively (***p < 0.001; *n* = 3; Student’s two-tailed *t* test). **f** Overexpression of FBXW2 induced G1-phase cell cycle arrest in PC3 cells. The PC3 cells were transfected with vector or HA-FBXW2, and cell cycle distributions were then analyzed by flow cytometry (**p < 0.01, ***p < 0.001; *n* = 3; Student’s two-tailed *t* test). **g** Effects of FBXW2 overexpression on the expression of cell progression-related proteins in PC3 cells. GAPDH levels served as the control for equal loading (***p < 0.001; *n* = 3; Student’s two-tailed *t* test)

FBXW2, acts as a tumor suppressor gene in lung cancer by directly degrading substrates or by regulating other E3 ligases [15, 18]. However, the biological function of FBXW2 in PCa is previously unknown. To study the function of FBXW2 involved in PCa, we first investigated FBXW2 levels in clinical samples. We found that both mRNA and protein of FBXW2 were significantly down-regulated in metastatic PCa (M-PCa) tissues than non-metastatic PCa (PCa) and prostatic hyperplasia (Con) (Fig. 1a). FBXW2 was strongly expressed in low-metastatic PCa cell lines (LNCaP and 22RV1) and human normal prostatic epithelial cell line (RWPE), whereas was weakly expressed in highly-metastatic PCa cell lines (PC3 and DU145) (Fig. 1b). To further confirm whether FBXW2 inhibits growth and metastasis of PCa, we transfected plasmids or siRNA of FBXW2 into PCa cells (Supplemental Fig. 1a and Supplemental Fig. 3a). Indeed, the in vitro experiments confirmed that overexpression of FBXW2 markedly suppressed cell abilities of proliferation, migration and invasion (Fig. 1c-e and Supplemental Fig. 1b–d). From three RNA interference of FBXW2 (#970, #1014 and #1174), we confirmed si-FBXW2-#1014 was the most efficient infection fragment (Supplemental Fig. 3). We further found si-FBXW2-#1014 significantly stimulated cell abilities of proliferation, migration, and invasion (Supplemental Fig. 4a–c). Further analysis showed that FBXW2 overexpression led to arrest at G1 phase (Fig. 1f and Supplemental Fig. 1e), which was further supported by the decrease of G1 phase proteins cyclin B1, cyclin D1, and cyclin E1 (Fig. 1g and Supplemental Fig. 1f). But FBXW2 had little influence on cell death (Supplemental Fig. 2). To further elucidate the mechanism by which FBXW2 suppressed cell growth and metastasis, after manipulation of FBXW2, we measured the levels of few related proteins. Consistent with in vitro experiments results, FBXW2 overexpression caused a decrease of N-cadherin and the increase of E-cadherin, p21 and p27 (Fig. 1g and Supplemental Fig. 1f), while FBXW2 knockdown (si-FBXW2-#1014) exhibited the opposite effect (Supplemental Fig. 4d). Taken together, these results strongly suggested that FBXW2 may as a tumor suppressor to suppress growth and metastasis of PCa cells.

### Augmented FBXW2 inhibits PCa tumor growth and attenuates osteolytic bone tumor tumorigenesis in vivo

**Fig. 2:**
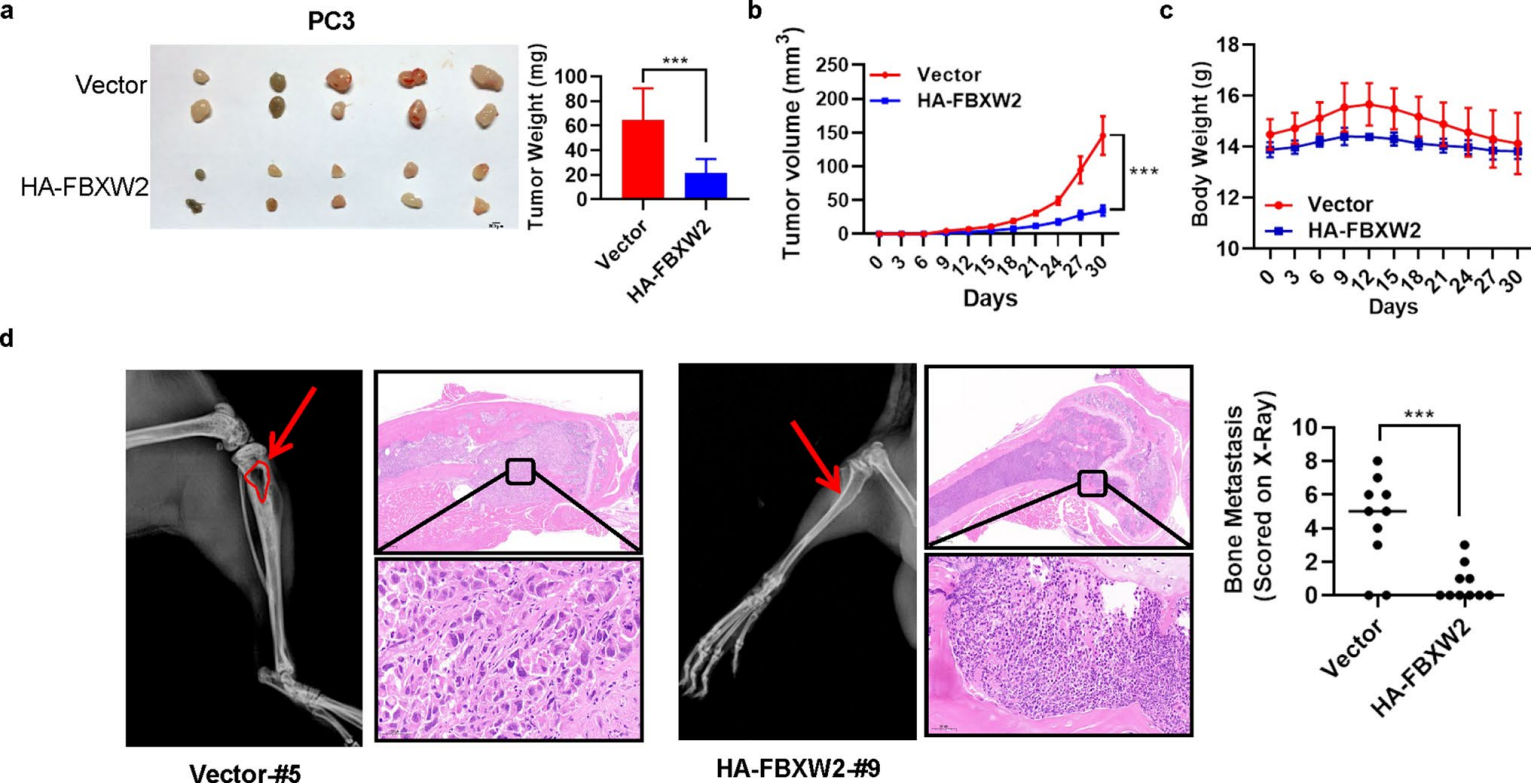
Augmented FBXW2 inhibits PCa tumor growth and attenuates osteolytic bone tumor tumorigenesis. **a–c** Overexpression of FBXW2 inhibited PCa tumor growth in vivo. PC3 cells stably expressing Vector and HA-FBXW2 (1 × 10^6^ cells) were inoculated s.c. in both flanks of nude mice. After indicated time, the tumors were harvested and photographed (**a**) (***p < 0.001; *n* = 5; Student’s two-tailed *t* test; Scale bars, 200 μm). The tumor growth was monitored twice a week for up to 30 days and growth curve plotted (**b**) (***p < 0.001; *n* = 5; two-way ANOVA). Body weight was measured and plotted (**c**) (ns, no significance; *n* = 5; two-way ANOVA). **d** Overexpression of FBXW2 attenuated osteolytic bone tumor tumorigenesis in vivo. 2.0 × 10^6^ PC3 stable cells (Vector or HA-FBXW2) were re-suspended in 100 µL PBS, and 10 µL of cell solution was slowly injected into NOD/SCID mice. Representative radiographical images of osteolytic bone tumor in the indicated tibia of the mice (left). Bars, 4 mm. Representative H&E-stained sections of the indicated tibia of the mice (right). Scale bar, 500 µm and 50 µm. The sum of bone metastasis scores in the tibia of the Vector or HA-FBXW2 mice groups (***p < 0.001; *n* = 10; Student’s two-tailed *t* test)

To test the tumor suppressor potential of FBXW2 in vivo, we performed a xenograft model by inoculating FBXW2 overexpression of PC3 stable cell line into both flanks of nude mice. We found that overexpression of FBXW2 significantly suppressed the in vivo tumor growth, decreased the tumor size and weight compared to the vector control (Fig. 2a–b), but had no effects on the mouse body weight (Fig. 2c). Meanwhile, to determine the effect of FBXW2 on bone metastasis of PCa in vivo, the FBXW2 overexpression of PC3 cells were inoculated into both tibial bone of the mice to monitor development of bone metastatic tumors. Interestingly, overexpression of FBXW2 did cause a significant reduction in osteolytic areas compared with control vector (Fig. 2d). Likewise, H&E staining also demonstrated that overexpression of FBXW2 dramatically reduced the tumor burden in bone. Collectively, the results indicated overexpression of FBXW2 could inhibit tumor growth of PCa and attenuate osteolytic bone destruction.

### FBXW2 is inversely correlated with EGFR in PCa, and regulates EGFR protein level

**Fig. 3:**
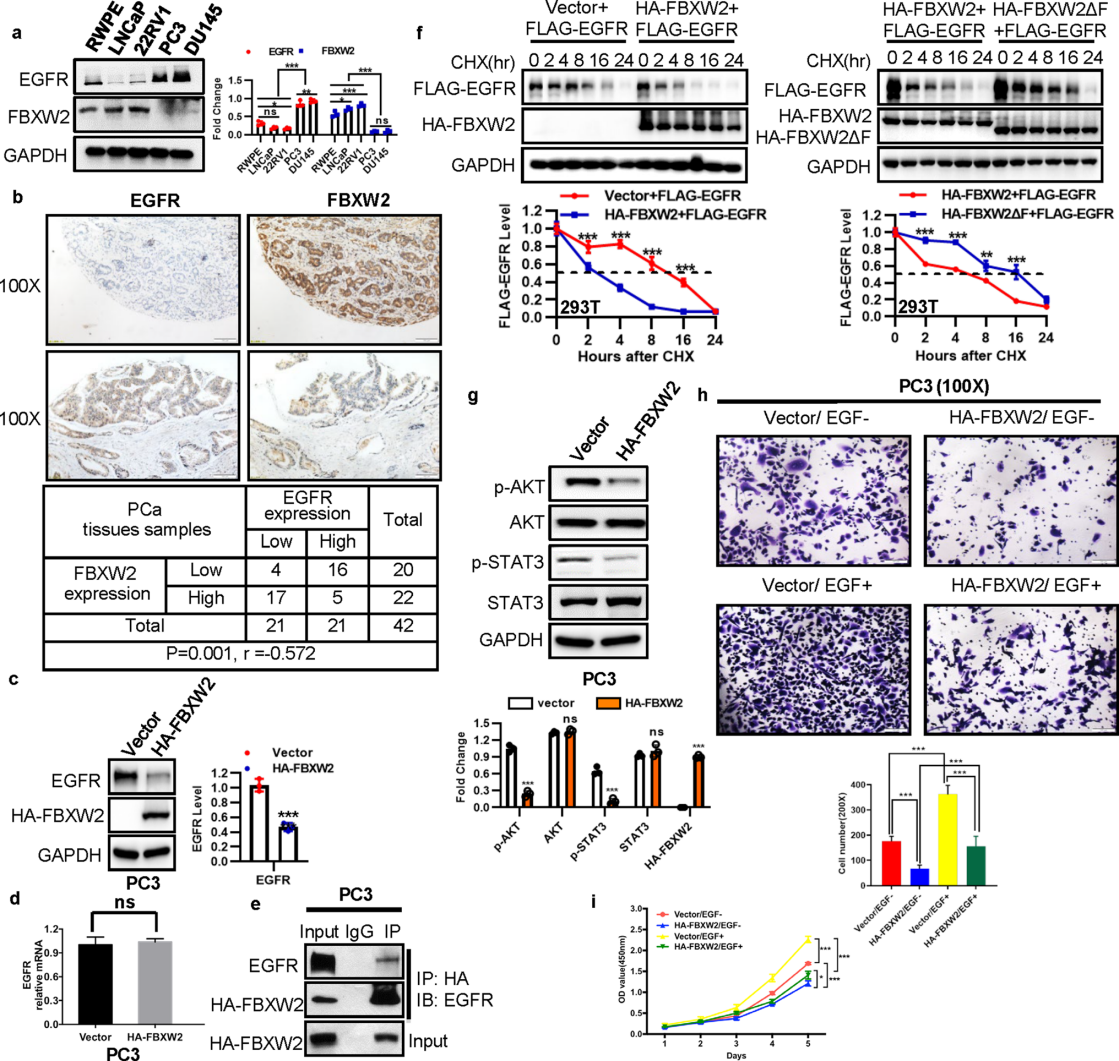
FBXW2 is inversely correlated with EGFR in PCa, and regulates EGFR protein level. **a** Inverse correlation at the protein levels of EGFR versus FBXW2 in PCa cell lines (*p < 0.05, **p < 0.01, ***p < 0.001; *n* = 3; one-way ANOVA). **b** Expression of FBXW2 and EGFR in PCa tissues. PCa tissue microarrays were stained with FBXW2 and EGFR, and then photographed (Scale bars, 100 μm). Association analysis of FBXW2 and EGFR in PCa tissues. Data were analyzed using SPSS software (*P* = 0.001, *n* = 42, Pearson’s test). **c**, **d** Overexpression of FBXW2 reduced EGFR protein level, but not EGFR mRNA levels. PC3 cells were transfected with vector or HA-FBXW2, and followed by IB (**c**) or qPCR (**d**) (***p < 0.001; *n* = 3; Student’s two-tailed t test). **e** FBXW2 could bind to endogenous EGFR: Cell lysates from PC3 cells were pulled down with anti-FBXW2 Abs, followed by IB with indicated Abs. **f** Overexpression of FBXW2, but not its ΔF mutant, shortened protein half-life of exogenous EGFR. After transfection with relevant plasmids for 48 h, 293 T cells were switched to fresh medium (10% FBS) containing cycloheximide (CHX) and incubated for indicated time periods before being harvested for IB. The band density was quantified using ImageJ software and plotted (**p < 0.01; ***p < 0.001; *n* = 3; two-way ANOVA). **g** Protein levels of EGFR downstream were markedly decreased by FBXW2 overexpression. PC3 cells were transfected with vector or HA-FBXW2, and followed by IB (ns, no significance, ***p < 0.001; *n* = 3; Student’s two-tailed *t* test). **h**, **i** Transfection of FBXW2 significantly abrogated invasion ability (**h**) and proliferation (**i**) caused by EGF. Scale bar: 100 µm (**h**). Transfected the vector control or plasmid expressing HA-FBXW2 for 48 h in PC3 cells were treated with or without EGF (10 ng/mL). The cell ability and invasion ability were detected by CCK-8 assay (*p < 0.1, ***p < 0.001; *n* = 3; two-way ANOVA) and transwell assays (***p < 0.001; *n* = 3; one-way ANOVA), respectively

FBXW2 has been confirmed to promote ubiquitylation and degradation of oncogenic proteins, such as SKP2 and β-catenin, resulting in suppression of tumor growth and metastasis of lung cancer [15, 18]. Also, we found FBXW2 inhibited tumor growth of PCa and attenuate osteolytic bone destruction. And dysregulation of EGFR plays a significant role in proliferation and survival of PCa as well as invasion and metastasis to the bone [5, 6, 21], but degradation mechanisms of EGFR is not fully understood. Based on that, we speculate if FBXW2 represses tumor growth and metastasis of PCa through regulating EGFR. To study the molecular mechanisms involved in the relationship between the two proteins, we first observed in general an inversely correlated expression pattern between FBXW2 and EGFR in multiple PCa cell lines (Fig. 3a). Next, the Pearson correlation analysis between FBXW2 and EGFR expressions in human PCa tumor samples showed that high EGFR levels had low FBXW2 expression (*P* = 0.001, *r* = − 0.572), and vice versa (Fig. 3b). When FBXW2 was transfected into PC3 and DU145 cells, a reduction of endogenous EGFR was detected (Fig. 3c and Supplemental Fig. 5a). On the contrary, we performed a siRNA-based knockdown (si-FBXW2-#1014) in 22RV1 cells and found that protein level of endogenous EGFR was increased (Supplemental Fig. 5a). Furthermore, neither FBXW2 overexpression nor depletion had any effect on the level of EGFR mRNA (Fig. 3d and Supplemental Fig. 5b), suggesting that EGFR protein level was negatively regulated by FBXW2. We next determined whether FBXW2 and EGFR bind to each other under physiological conditions. Indeed, using immunoprecipitation (IP), we detected endogenous EGFR in FBXW2 immuno-precipitants (Fig. 3e), indicating the physical interaction between EGFR and FBXW2. To further test whether FBXW2 regulates the stability of EGFR, we next determined the protein half-life of EGFR upon manipulation of FBXW2 in the presence of cyclohexamide (CHX) to block new protein synthesis. Indeed, wile-type FBXW2 ectopic expression significantly shortened half-life of exogenous EGFR protein in 293 T cells, but overexpression of FBXW2-ΔF mutant, which is a dominant-negative mutant that can bind to the substrates but fails to recruit the other SCF ubiquitin ligase components [15], had no effect on it (Fig. 3f). Whereas transfection of siRNA targeting FBXW2 (si-FBXW2-#1014) extended protein EGFR half-life, leading to its stabilization (Supplementary Fig. 5c). STAT3 and AKT proteins are known downstream targets of EGFR as well as promoters of tumor growth [22, 23]. We further found that the phosphorylation of STAT3 and AKT were markedly decreased by FBXW2 overexpression, while FBXW2 depletion caused their accumulation (Fig. 3g and Supplemental Fig. 5a). A similar dose-dependent reduction of endogenous EGFR, p-AKT, and p-STAT3 was also detected when FBXW2 was transfected (Supplemental Fig. 5d). Using IB and IHC staining, we also observed that the levels of EGFR, p-AKT, and p-STAT3 were down-regulated in FBXW2-overexpressing tumor tissues of mice model, whereas p21 and p27 were enhanced (Supplemental Fig. 6). We further sought to determine whether FBXW2 can block EGF-induced biological effects. Treated with EGF significantly stimulated invasion and cell proliferation (Fig. 3h–i and Supplemental Fig. 7). This effect could be completely abrogated when cells were transfected FBXW2. Collectively, the results from both in vitro cell culture and in vivo mice models coherently demonstrated that FBXW2 overexpression significantly attenuated cell proliferation and metastasis by suppressing EGFR and its downstream, which was dependent on the structural integrity of F-box domain of FBXW2.

### FBXW2 binds to EGFR via its consensus degron motif, and ubiquitylates EGFR

**Fig. 4:**
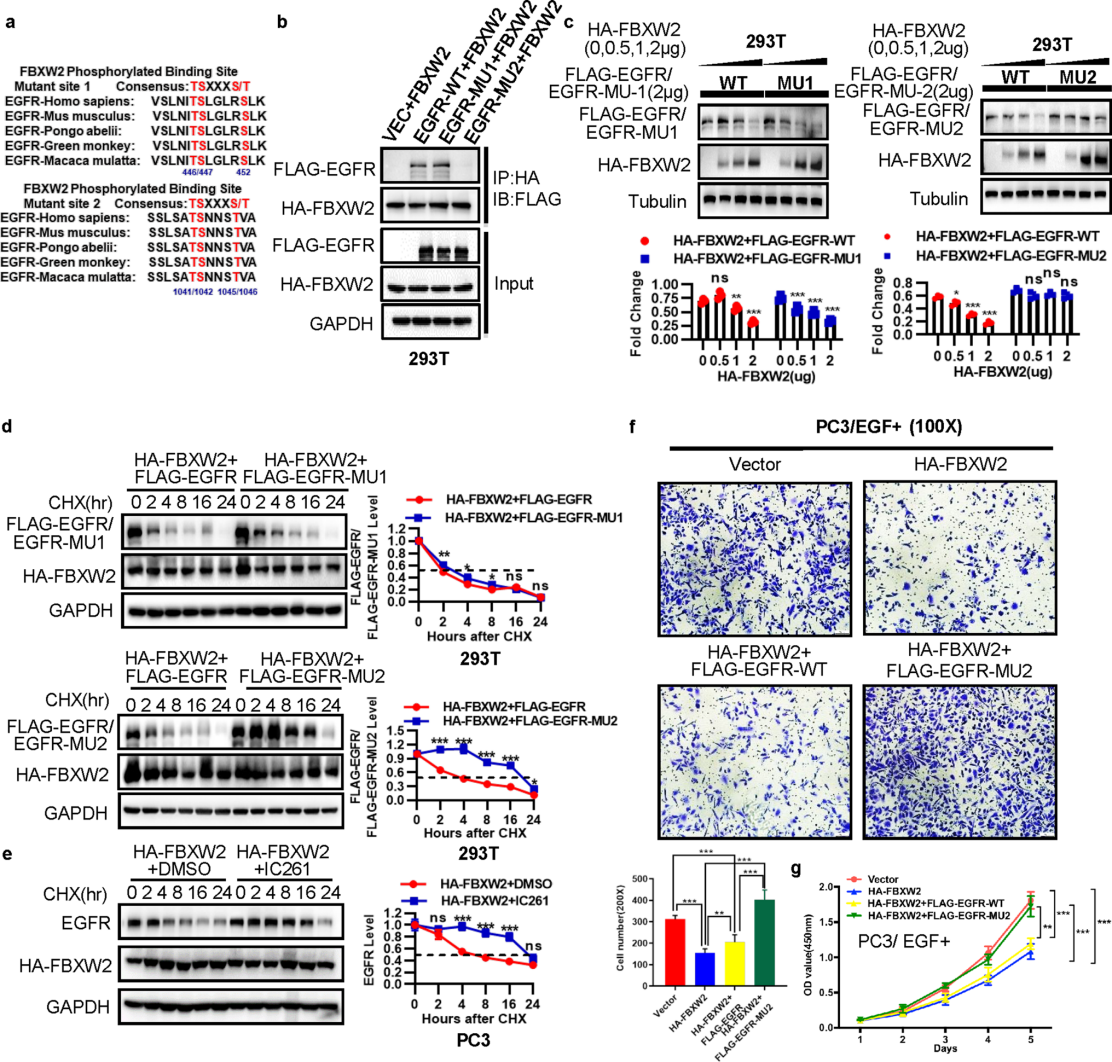
FBXW2 binds to EGFR via its consensus degron motif, and degrades EGFR. **a** Two possible binding sites on EGFR, MU1 (446/447, 452) and MU2 (1041/1042, 1045/1046). **b** Loss of FBXW2-EGFR binding site in mutant: EGFR or its mutants was co-transfected with HA-FBXW2, followed by IP with HA Abs and IB. **c** Overexpression of FBXW2 reduced basal level of EGFR and EGFR-MU1 in a dose manner, but not EGFR-MU2. (ns, no significance, *p < 0.05, **p < 0.01, ***p < 0.001; *n* = 3; one-way ANOVA). **d** FBXW2 shortened the protein half-life of EGFR and EGFR-MU1, but not EGFR-MU2. Stable 293 T cells were incubated with CHX for indicated time periods and harvested for IB (ns, no significance, *p < 0.05, **p < 0.01, ***p < 0.001; *n* = 3; two-way ANOVA). **e** CK1 kinase mediated EGFR phosphorylation at FBXW2 binding motif. PC3 cells were transfected HA-FBXW2, and treated with or without CK1 inhibitor IC261 (10 mM), and harvested for IB. GAPDH levels served as the control for equal loading (ns, no significance, ***p < 0.001; *n* = 3; two-way ANOVA). **f**, **g** Under EGF stimulation, transfected FBXW2 alone or co-transfection with wild-type EGFR suppressed and invasion (**f**) and cell proliferation (**g**) in PC3 cells, but was abrogated by simultaneous transfection of EGFR-MU2. Scale bar:100 µm (**f**). Transfected HA-FBXW2 alone or in combinations with FLAG-EGFR (WT versus MU2) into PC3 cells and then treated with EGF (10 ng/mL). The cell proliferation and invasion ability were detected by CCK-8 assay (**p < 0.01, ***p < 0.001; *n* = 3; two-way ANOVA) and transwell assays (**p < 0.01, ***p < 0.001; *n* = 3; one-way ANOVA), respectively

It has been confirmed that substrate ubiquitination and degradation of FBXW2 depend on the presence of the degron motif TSXXXS/T in the substrate protein sequence that can be specifically recognized by FBXW2 [15, 18]. Comparing the sequence of EGFR with FBXW2, we found that EGFR had two evolutionarily conserved binding sites (TSLGLRS and TSNNST) similar to FBXW2 degradation motif on codons 446–452 and 1041–1046. We further generated two EGFR mutants on FBXW2 degron site by changing ‘TSLGLRS’ to ‘AALGLRA’ and ‘TSNNST’ to ‘AANNAA’, which were named as ‘EGFR-MU1’ and ‘EGFR-MU2’ (Fig. 4a), respectively. Transfection followed by IP/IB assay revealed that wild-type EGFR (EGFR-WT) and EGFR-MU1 mutant bound more effectively to FBXW2, whereas EGFR-MU2 failed to do so (Fig. 4b). Furthermore, FBXW2 no longer had any effect on the basal level of ectopically expressed EGFR-MU2 mutant (Fig. 4c), nor on its half-life (Fig. 4d), indicating that the stability of EGFR, negatively regulated by FBXW2, was dependent on the FBXW2 degron motif (1041/1042 and 1045/1046). It is well-established that in most cases, phosphorylation is prerequisite for a substrate to bind to a F-box protein for targeting ubiquitylation and degradation by the SCF ubiquitin ligases [18, 24]. To identify the potential kinase(s) that would mediate EGFR phosphorylation at the FBXW2 degron motif (TSXXXS/T), we searched computer database (GSP 3.0 http://gps.biocuckoo.org) for consensus kinase binding site, and identified CK1 (Casein kinase) as a candidate with the highest score (Table 1). We, therefore, added CK1 inhibitor IC261 and found that overexpression of FBXW2-induced degradation of endogenous EGFR was largely blocked by CK1 inhibition (Fig. 4e), indicating that CK1 kinase appeared to mediate EGFR phosphorylation at the FBXW2 binding motif. Moreover, under the condition with EGF stimulation, overexpression of FBXW2 inhibited cell growth and invasion by promoting wild-type EGFR (EGFR-WT) degradation, and this function was remarkably blocked when EGFR-MU2 mutant was used (Fig. 4f, g and Supplemental Fig. 8), suggesting that growth- and invasion-inhibiting effect of FBXW2 was mediated by targeting EGFR degradation via its consensus degron motif, resulting in repression of EGF function.

**Table 1:** Putative kinases for FBXW2 phosphorylation at the EGFR binding motif (TSNNST)

Position	Code	Kinase	Peptide	Score	Cutoff
9	T	CAMK	SATSNNS***T***VACI***	2.08	1.749
2	S	CK1	L***S***ATSNNST******	13.174	7.074
4	T	CK1	LSA***T***SNNSTVA****	10.576	7.074
5	S	CK1	LSAT***S***NNSTVAC***	12.035	7.074
8	S	CK1	LSATSNN***S***TVACI**	11.229	7.074
9	T	CK1	SATSNNS***T***VACI***	11.715	7.074
2	S	TKL	L***S***ATSNNST******	4.746	4.148
4	T	TKL	LSA***T***SNNSTVA****	12.197	4.148
5	S	TKL	LSAT***S***NNSTVAC***	5.296	4.148
9	T	TKL	SATSNNS***T***VACI***	6.31	4.148
2	S	AGC/DMPK	L***S***ATSNNST******	4.232	2.427
4	T	AGC/DMPK	LSA***T***SNNSTVA****	3.942	2.427
5	S	AGC/DMPK	LSAT***S***NNSTVAC***	2.536	2.427
8	S	AGC/DMPK	LSATSNN***S***TVACI**	3.449	2.427
2	S	AGC/GRK	L***S***ATSNNST******	14.034	7.991
4	T	AGC/GRK	LSA***T***SNNSTVA****	13.069	7.991
5	S	AGC/GRK	LSAT***S***NNSTVAC***	14.216	7.991
8	S	AGC/GRK	LSATSNN***S***TVACI**	18.259	7.991
9	T	AGC/GRK	SATSNNS***T***VACI***	22.457	7.991
2	S	AGC/PKC	L***S***ATSNNST******	2.552	1.416
4	T	AGC/PKC	LSA***T***SNNSTVA****	1.742	1.416
5	S	AGC/PKC	LSAT***S***NNSTVAC***	1.965	1.416
9	T	AGC/PKC	SATSNNS***T***VACI***	1.572	1.416
9	T	Atypical/PDHK	SATSNNS***T***VACI***	5.548	4.981
8	S	CAMK/MAPKAPK	LSATSNN***S***TVACI**	9.753	8.972
2	S	CAMK/PHK	L***S***ATSNNST******	9.346	3.785
4	T	CAMK/PHK	LSA***T***SNNSTVA****	6.038	3.785
5	S	CAMK/PHK	LSAT***S***NNSTVAC***	5.692	3.785
8	S	CAMK/PHK	LSATSNN***S***TVACI**	4.5	3.785
9	T	CAMK/PHK	SATSNNS***T***VACI***	5.885	3.785
5	S	CK1/CK1	LSAT***S***NNSTVAC***	6.546	3.998
8	S	CK1/CK1	LSATSNN***S***TVACI**	6.992	3.998
2	S	CK1/VRK	L***S***ATSNNST******	7.5	0.977
4	T	CK1/VRK	LSA***T***SNNSTVA****	8.8	0.977
5	S	CK1/VRK	LSAT***S***NNSTVAC***	7.6	0.977
8	S	CK1/VRK	LSATSNN***S***TVACI**	2.1	0.977
9	T	CK1/VRK	SATSNNS***T***VACI***	1.6	0.977
2	S	TKL/RIPK	L***S***ATSNNST******	2.667	1.75
4	T	TKL/RIPK	LSATSNNSTVA****	2.667	1.75
8	S	TKL/RIPK	LSATSNN***S***TVACI**	4	1.75
2	S	TKL/STKR	L***S***ATSNNST******	6.5	5.609
5	S	TKL/STKR	LSAT***S***NNSTVAC***	5.938	5.609
8	S	TKL/STKR	LSATSNN***S***TVACI**	6.438	5.609
2	S	AGC/GRK/BARK	L***S***ATSNNST******	11.233	4.78
4	T	AGC/GRK/BARK	LSA***T***SNNSTVA****	11.372	4.78
5	S	AGC/GRK/BARK	LSAT***S***NNSTVAC***	9.581	4.78
8	S	AGC/GRK/BARK	LSATSNN***S***TVACI**	9.767	4.78
9	T	AGC/GRK/BARK	SATSNNS***T***VACI***	17.209	4.78
4	T	AGC/GRK/GRK	LSA***T***SNNSTVA****	3.906	3.858
2	S	AGC/PKC/PKCa	L***S***ATSNNST******	6.178	4.803
5	S	AGC/PKC/PKCa	LSAT***S***NNSTVAC***	5.578	4.803
2	S	AGC/RSK/MSK	L***S***ATSNNST******	4.741	4.025
2	S	CAMK/CAMK1/CAMK4	L***S***ATSNNST******	3.542	2.206
4	T	CAMK/CAMK1/CAMK4	LSA***T***SNNSTVA****	3.208	2.206
9	T	CAMK/CAMK2/CAMK2B	SATSNNS***T***VACI***	8.718	8.277
2	S	CAMK/CAMKL/AMPK	L***S***ATSNNST******	2.568	1.777
4	T	CAMK/CAMKL/LKB	LSA***T***SNNSTVA****	2.9	2.821
9	T	CAMK/CAMKL/MELK	SATSNNS***T***VACI***	1.944	1.36
4	T	CAMK/CAMKL/PASK	LSA***T***SNNSTVA****	6	5.767
2	S	CAMK/MAPKAPK/MK5	L***S***ATSNNST******	11.727	9.696
2	S	CAMK/MAPKAPK/MNK	L***S***ATSNNST******	10	8.025
2	S	CAMK/PKD/PRKD1	L***S***ATSNNST******	2.115	1.5
8	S	CK1/CK1/CK1-A	LSATSNN***S***TVACI**	3.947	3.887
9	T	CK1/CK1/CK1-A	SATSNNS***T***VACI***	4.263	3.887
2	S	CK1/CK1/CK1-D	L***S***ATSNNST******	7.861	4.344
4	T	CK1/VRK/VRK1	LSA***T***SNNSTVA****	5.2	3.159
5	S	CK1/VRK/VRK1	LSAT***S***NNSTVAC***	4.1	3.159
2	S	CK1/VRK/VRK2	L***S***ATSNNST******	24.5	5.225
***4***	***T***	***CK1/VRK/VRK2***	LSATSNNSTVA****	***28.75***	***5.225***
***5***	***S***	***CK1/VRK/VRK2***	LSATSNNSTVAC***	***27.5***	***5.225***
***8***	***S***	***CK1/VRK/VRK2***	LSATSNNSTVACI**	***17.75***	***5.225***
***9***	***T***	***CK1/VRK/VRK2***	SATSNNSTVACI***	***18.25***	***5.225***
4	T	CMGC/CDK/CDK9	LSA***T***SNNSTVA****	5.45	4.647
8	S	TKL/IRAK/IRAK4	LSATSNN***S***TVACI**	7.2	6.78
2	S	TKL/MLK/MLK	L***S***ATSNNST******	3.909	1.5
4	T	TKL/MLK/MLK	LSA***T***SNNSTVA****	1.727	1.5
5	S	TKL/STKR/STKR1	LSAT***S***NNSTVAC***	8.625	8.6
4	T	TKL/STKR/STKR2	LSA***T***SNNSTVA****	1.75	1.344
2	S	AGC/GRK/BARK/BARK1	L***S***ATSNNST******	15.405	5.269
4	T	AGC/GRK/BARK/BARK1	LSA***T***SNNSTVA****	8.405	5.269
5	S	AGC/GRK/BARK/BARK1	LSAT***S***NNSTVAC***	10.952	5.269
8	S	AGC/GRK/BARK/BARK1	LSATSNN***S***TVACI**	21	5.269
9	T	AGC/GRK/BARK/BARK1	SATSNNS***T***VACI***	28.595	5.269
2	S	AGC/GRK/GRK/GRK	L***S***ATSNNST******	12.778	9.35
4	T	AGC/GRK/GRK/GRK	LSA***T***SNNSTVA****	10.278	9.35
2	S	AGC/PKC/PKCa/PRKCA	L***S***ATSNNST******	11.051	7.372
4	T	AGC/PKC/PKCa/PRKCA	LSA***T***SNNSTVA****	13.889	7.372
5	S	AGC/PKC/PKCa/PRKCA	LSAT***S***NNSTVAC***	18.152	7.372
8	S	AGC/PKC/PKCa/PRKCA	LSATSNN***S***TVACI**	14	7.372
9	T	AGC/PKC/PKCa/PRKCA	SATSNNS***T***VACI***	13.348	7.372
2	S	AGC/RSK/RSKp70/RPS6KB1	L***S***ATSNNST******	2.88	2.548
2	S	AGC/RSK/RSKp90/RPS6KA1	L***S***ATSNNST******	4.375	2.462
5	S	Atypical/PDHK/PDHK/PDK1	LSAT***S***NNSTVAC***	7.459	4.74
9	T	Atypical/PDHK/PDHK/PDK1	SATSNNS***T***VACI***	6.568	4.74
4	T	CAMK/CAMKL/AMPK/AMPKA1	LSA***T***SNNSTVA****	7.75	7.125
5	S	CAMK/CAMKL/AMPK/PRKAB1	LSAT***S***NNSTVAC***	5.667	2.525
4	T	CAMK/CAMKL/LKB/STK11	LSA***T***SNNSTVA****	2.793	2.793
2	S	CAMK/CAMKL/QIK/SIK1	L***S***ATSNNST******	10.688	4.074
4	T	CAMK/CAMKL/QIK/SIK1	LSA***T***SNNSTVA****	8.312	4.074
5	S	CAMK/CAMKL/QIK/SIK1	LSAT***S***NNSTVAC***	7.188	4.074
8	S	CAMK/CAMKL/QIK/SIK1	LSATSNN***S***TVACI**	4.25	4.074
2	S	CAMK/MAPKAPK/MK5/MAPKAPK5	L***S***ATSNNST******	11.727	9.696
8	S	CAMK/MAPKAPK/MNK/MNK2	LSATSNN***S***TVACI**	16.429	9.344
9	T	CK1/CK1/CK1-A/CSNK1A1	SATSNNS***T***VACI***	3.421	2.971
4	T	CK1/CK1/CK1-D/CK1e	LSA***T***SNNSTVA****	11.353	6.526
9	T	CK1/CK1/CK1-D/CK1e	SATSNNS***T***VACI***	6.529	6.526
9	T	CMGC/CDK/CDK7/CDK7	SATSNNS***T***VACI***	0.667	0.406
4	T	CMGC/MAPK/p38/MAPK13	LSA***T***SNNSTVA****	5	4.706
5	S	CMGC/MAPK/p38/MAPK13	LSAT***S***NNSTVAC***	5.765	4.706
2	S	TKL/MLK/MLK/MLK1	L***S***ATSNNST******	3.333	2.6
8	S	TKL/MLK/MLK/MLK1	LSATSNN***S***TVACI**	3	2.6
8	S	TKL/MLK/MLK/MLK3	LSATSNN***S***TVACI**	3.75	3.6
2	S	TKL/STKR/STKR1/BMPR1B	L***S***ATSNNST******	5.667	5.158
4	T	TKL/STKR/STKR2/TGFbR2	LSA***T***SNNSTVA****	1.75	1.344

**Position**: The position of the site that is predicted to be phosphorylated. **Code**: The residue that is predicted to be phosphorylated. **Kinase**: The regulatory kinase that is predicted to phosphorylate the site. **Peptide**: The predicted phosphopeptide with seven amino acids upstream and seven amino acids downstream around the modified residue. **Score**: The value calculated by GPS algorithm (http://gps.biocuckoo.org) to evaluate the potential of phosphorylation. The higher the value, the more potential the residue is phosphorylated. **Cutoff**: The cutoff value under the threshold. Different threshold means different precision, sensitivity, and specificity. * represents amino acids not shown

**Fig. 5:**
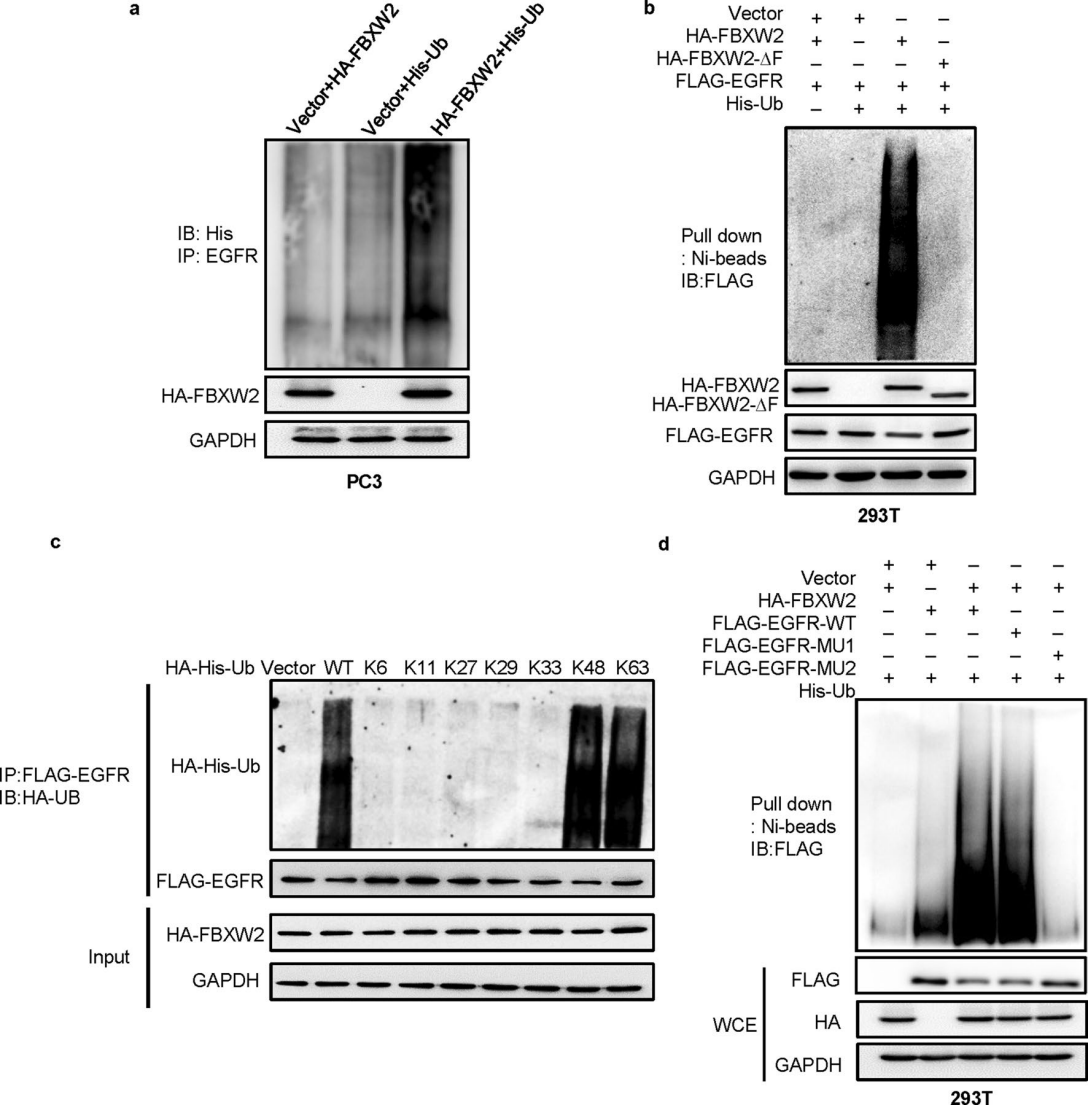
FBXW2 promotes EGFR ubiquitylation via its consensus degron motif (TSNNST) on EGFR for subsequent degradation. **a** FBXW2 promoted ubiquitylation of EGFR in vivo: PC3 cells were transfected with indicated plasmids, lysed under denatured condition at 6 M guanidinium solution, followed by Ni-beads pull-down. **b** FBXW2, but not its ΔF mutant, promoted ubiquitylation of EGFR in vivo: 293 T cells were transfected with indicated plasmids, lysed under denatured condition at 6 M guanidinium solution, followed by Ni-beads pull-down. **c** EGFR was conjugated with lysine (K) 48- or 63-linked polyubiquitin chains: 293 T cells transfected with different ubiquitin mutants were co-overexpressed both HA-FBXW2 and FLAG-EGFR. After 48 h transfection, cells were treated with MG132 for 4 h followed by IP and IB. **d** FBXW2 promoted ubiquitylation of wild-type EGFR and EGFR-MU1, not EGFR-MU2 in vivo: 293 T cells were transfected with indicated plasmids, lysed under denatured condition at 6 M guanidinium solution, followed by Ni-beads pull-down. GAPDH levels served as the control for equal loading

We next investigated, using classic in vivo ubiquitylation assays, whether FBXW2 promotes the ubiquitylation of EGFR for enhanced degradation. Indeed, the in vivo ubiquitylation assay showed that FBXW2 significantly promoted ubiquitylation of endogenous EGFR (Fig. 5a). Wild-type FBXW2 also promoted ubiquitylation of exogenous EGFR and this activity was remarkably reduced when FBXW2-ΔF mutant was used (Fig. 5b). To determine which lysine (K)-linked polyubiquitin chain conjugated with EGFR, we overexpressed several ubiquitin mutants (K6, K11, K27, K29, K33, K48, and K63). We found that EGFR was conjugated with lysine (K) 48- or 63-linked polyubiquitin chains (Fig. 5c). Furthermore, FBXW2 only promoted ubiquitylation of wild-type EGFR (EGFR-WT) and EGFR-MU1 mutant, but not EGFR-MU2 mutant (Fig. 5d). Taken together, we concluded that FBXW2 acted as a ubiquitin ligase that bond to EGFR via its consensus degron motif (1041/1042 and 1045/1046)*,* and promoted ubiquitylation and subsequent degradation of EGFR to shorten its protein half-life.

## Discussion

What the biological functions of FBXW2 in PCa and whether FBXW2 targets other substrates to involve in progression of PCa remain unknown. Herein, we reported that FBXW2 acted as a novel tumor suppressor in PCa by promoting the ubiquitylation and degradation of the oncogenic protein EGFR with following lines of supporting evidence: (1) FBXW2 is down-regulated in highly-metastasis PCa cells and tissues; (2) Enhanced FBXW2 significantly attenuates growth and metastasis of PCa models in vitro and in vivo; (3) EGFR is overexpressed in PCa cells, which has been demonstrated to contribute to growth and metastatic of PCa [27]. And EGFR protein level and its half-life can be extended or decreased by FBXW2 depletion or overexpression, respectively, but FBXW2 dominant-negative mutant has no effect on it; (4) FBXW2 binds to EGFR via its consensus degron motif (TSNNST), and promotes EGFR ubiquitylation and degradation; (5) The tumor growth- and invasion- inhibiting effect of FBXW2 in PCa is casually mediated by down-regulating EGFR and its downstream targets; (6) FBXW2 blocks EGF-induced biological effects. Hence, we have found that FBXW2 is a tumor suppressor of PCa, which down-regulates EGFR downstream by promoting EGFR ubiquitination and degradation, resulting in repression of PCa cell proliferation and metastasis.

The F-box protein is a substrate recognition subunit of the SKP1-Cullin 1-F-box protein (SCF) E3 ligase complex, and plays a key role in multiple cellular processes through ubiquitylation and subsequent degradation of the target protein. F-box protein dysfunction plays an important role in the occurrence and development of PCa. The F-box protein SKP2 was first discovered as the E3 kinase of the p27 [23]. Subsequent studies reported that high expression of SKP2 promoted cell proliferation, invasion and shortened the survival of PCa patients [25, 26]. FBXW2 was originally identified as a ubiquitin ligase for polyubiquitination and degradation of GCM1, which suppressed placental cell migration and invasion [22, 27, 28]. Our recent study showed that FBXW2 was a tumor suppressor in lung cancer by promoting the ubiquitylation of SKP2 and β-catenin [15, 18]. Here, consistent with our previous study in lung cancer, we found that FBXW2 was a tumor suppressor in PCa by inhibiting the cell growth and metastasis.

Our study further identified that the tumor suppressing function of FBXW2 was through its ubiquitylation and degradation of EGFR and hence targeting EGFR in PCa will be a good therapeutic strategy. Abnormal activation of EGFR in PCa contributed to metastatic progression and poor prognosis of PCa [29]. EGFR reduced the tumor suppressor miR-1 and activated the oncogene TWIST1 to promote the progression of PCa and bone metastasis [5]. Cholesterol-rich Lipid Rafts in PCa cells promoted cell survival by activating the EGFR/PI3K/AKT pathway [23]. Furthermore, high expression of EGFR was specifically correlated with the high-grade stages and high risk for prostate-specific antigen recurrence [30]. Targeting EGFR alone [31] or combination with the conventional cytotoxic agents [32] or castration treatment [33] has shown that optimistic tumor growth inhibition in in vitro cell lines and in vivo xenograft models of PCa. Even though, the single-agent trials with the EGFR inhibitors, including gefitinib [34] and erlotinib [35, 36] or panitumumab [37] have not achieved any significant PSA response. These disappointing clinical trials may be explained by the well-known fact that EGFR inhibition in an unselected patient population is not a helpful approach. Indeed, inhibition of EGFR with cetuximab showed that significantly improved PFS in PCa patients with overexpression of EGFR and persistent activity of PTEN [38]. Consistent with this finding, we speculated that it will be more beneficial to the patients if EGFR was targeted in a selected group of PCa patients with low-expressed FBXW2.

Our study also raised a question. It is well known that the biological functions of an E3 ligase might be mediated by different substrates. Besides the newly-identified EGFR, the tumor growth and metastasis suppression effects of FBXW2 in PCa might be induced through other potential substrates of FBXW2, like SKP2 [18]. We will continue to pursue whether both SKP2 and EGFR are simultaneously accumulated in FBXW2-low-expressed PCa and whether double targeting SKP2 and EGFR will be more effective than single agent of each.

**Fig. 6:**
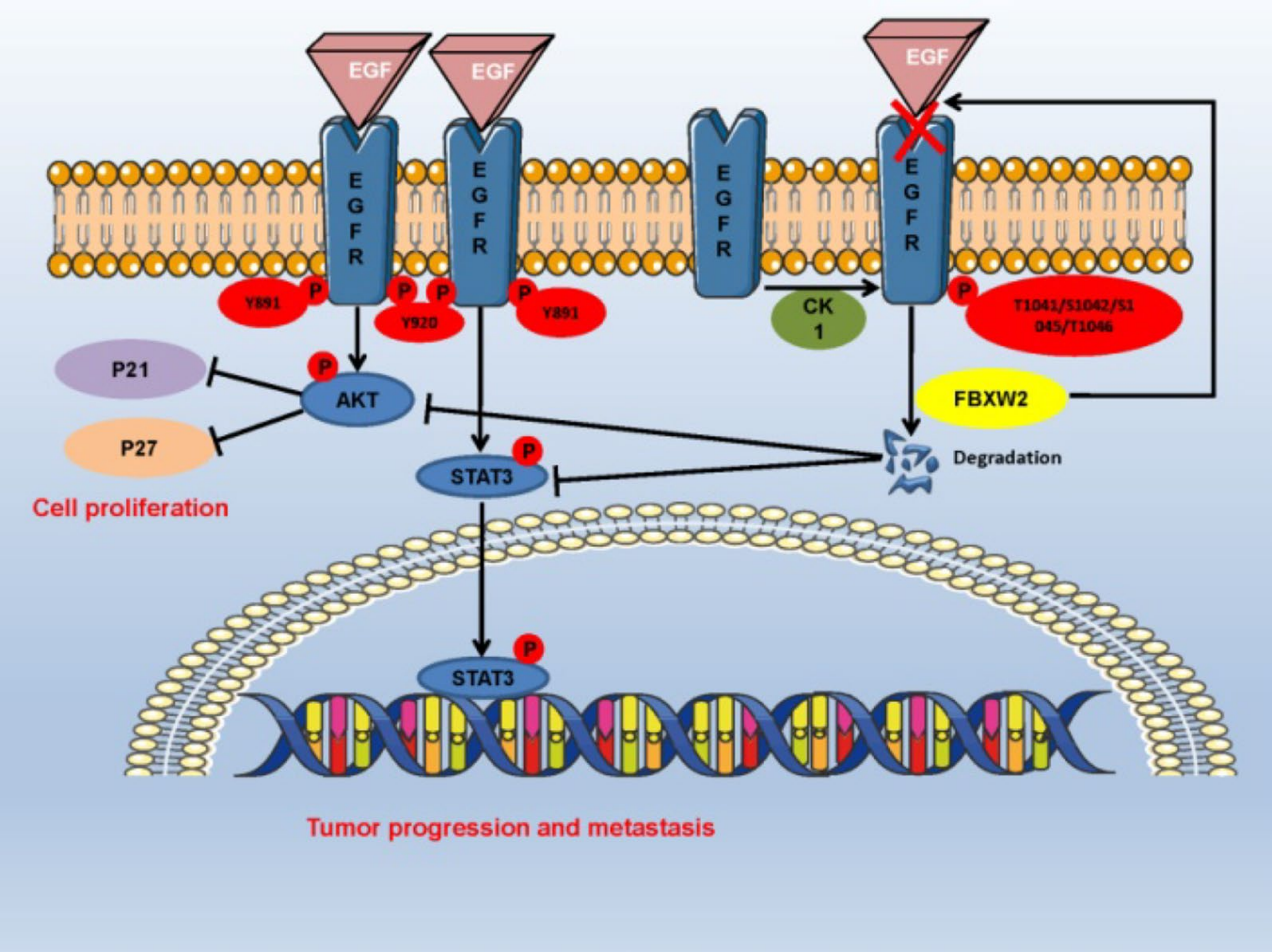
Schematic model for FBXW2-induced EGFR degradation inhibiting growth and metastasis of PCa cells. During tumorigenesis, FBXW2, as a tumor suppressor, promotes ubiquitylation and degradation of EGFR, leading to repression of EGFR downstream, eventually to control growth and metastasis of PCa cells

Collectively, our study reveals that FBXW2 is a novel E3 ligase of oncogenic protein EGFR for targeting ubiquitination and degradation, and results in repressing EGFR downstream, which inhibits PCa cell proliferation and metastasis (Fig. 6). These findings have motivated the development of a clinical trial to test whether targeting EGFR is effective in the selected patients with low-expressed FBXW2.

### Supplementary Information

Below is the link to the electronic supplementary material.

Supplementary file1 (DOCX 22 KB)

Supplementary file2 (PDF 1838 KB)

Supplementary file3 (PDF 2553 KB)

## Data Availability

All data generated or analyzed during this study are included in this published article.

## Abbreviations

PCa: Prostate cancer
PSA: Prostate-specific antigen
c-Cbl: Casitas Blineage lymphoma
FBXW2: F-box and WD-repeat domain-containing 2
SCF: SKP1-Cullin1-F-box protein
CRL1: Cullin-RING ligase 1
RBX1: Ring box protein-1
GCM1: Glial cell missing homolog 1
SKP2: S phase kinase-associated protein 2
CK1: Casein kinase
FBS: Fetal bovine serum
CCK-8: Cell counting Kit-8
OD: Optical density
eGFP: Enhanced green fluorescence protein
IB: Immunoblotting
IP: Immunoprecipitation
CHX: Cycloheximide
Ni–NTA: Nickel–nitrilotriacetic acid
IHC: Immunohistochemistry
HRP: Horseradish peroxidase
M-PCa: Metastatic prostate cancer
