# Supplementary material for: FBXW2 inhibits prostate cancer proliferation and metastasis via promoting EGFR ubiquitylation and degradation
Source: Cell Mol Life Sci. 2022 May 2;79(5):268. doi: 10.1007/s00018-022-04320-3 (PMC9061686; doi:10.1007/s00018-022-04320-3)
Supplement: Supplementary file 1 — Supplementary file1 (DOCX 22 KB) [file 18_2022_4320_MOESM1_ESM.docx]

**Supplementary figure legends**

**Supplementary Figure. 1 Overexpression of FBXW2 expression inhibits cancer growth and metastasis in PCa cells.**

**a** Transfected the vector control or plasmid expressing HA-FBXW2 in PC3 and DU145 cells, followed by IB. **b-d** Overexpression of FBXW2 in DU145 cells inhibited cell proliferation **(b)**, migration **(c)** and invasion **(d)**. Scale bar: 200µm (c) and 100µm (d). Cell viability was detected with Cell Counting Kit-8 (CCK-8) assay (****p* < 0.001; n = 3; two-way ANOVA). The cell migration ability and invasion ability were determined using the wound-healing and transwell assays, respectively (****p* < 0.001; n = 3; Student’s two-tailed t test). **e** Overexpression of FBXW2 induced G1-phase cell cycle arrest in DU145 cells. The PC3 cells were transfected with vector or HA-FBXW2, and cell cycle distributions were then analyzed by flow cytometry (***p* < 0.01; n = 3; Student’s two-tailed t test). **f** Effects of FBXW2 overexpression on the expression of cell progression-related proteins in DU145 cells. GAPDH levels served as the control for equal loading (****p* < 0.001; n = 3; Student’s two-tailed t test).

**Supplementary Figure. 2 Overexpression of FBXW2 in PCa cells has not effects on apoptosis.**

**a** Overexpression of FBXW2 failed to induce apoptosis in PCa cells. Apoptosis in PC3 and DU145 cells with transfected vector or HA-FBXW2 examined by flow cytometry (ns, no significance; n = 3; Student’s two-tailed t test). **b** Effects of FBXW2 overexpression on the expression of apoptosis-related proteins in PCa cells. The transfected PC3 and DU145 cells were harvested, and followed by IB. GAPDH levels served as the control for equal loading (ns, no significance; n = 3; Student’s two-tailed t test).

**Supplementary Figure. 3 FBXW2 silencing enhances cell abilities of proliferation and invasion in PCa cells.**

**a** 22RV1 and LNCaP cells were transfected with siRNA targeting FBXW2 (#970, #1014, #1174), along with scrambled siRNA, followed by IB. GAPDH levels served as the control for equal loading (ns, no significance, ***p* < 0.01, ****p* < 0.001; n = 3; Student’s two-tailed t test). **b** FBXW2 silencing promoted cancer cell proliferation. Transfected with either control RNAi, or RNAi targeting FBXW2 (#970, #1014, #1174), the cell viability was detected with CCK-8 assay (ns, no significance; **p* < 0.05; ***p* < 0.01; n = 3; two-way ANOVA). **c** FBXW2 silencing stimulated cancer cell invasion. 22RV1 and LNCaP cells were transfected with targeted siRNA, and followed by the transwell assays (ns, no significance; ****p* < 0.001; n = 3; Student’s two-tailed t test). Scale bar: 50µm.

**Supplementary Figure. 4 Knockdown of FBXW2 (si-FBXW2-#1014) growth and metastasis of PCa cells.**

**a-c** Knockdown of FBXW2 (si-FBXW2-#1014) enhanced cell abilities of proliferation **(a)**, migration **(b)** and invasion **(c)** of 22RV1 cells. Scale bar: 100µm (b and c). Cell viability was detected with Cell Counting Kit-8 (CCK-8) assay (***p* < 0.01; n = 3; two-way ANOVA). The cell migration ability and invasion ability were determined using the wound-healing and transwell assays, respectively (****p* < 0.001; n = 3; Student’s two-tailed t test). **d** Effects of FBXW2 depletion on the expression of cell progression-related proteins in 22RV1 cells. GAPDH levels served as the control for equal loading (**p* < 0.05; ***p* < 0.01; ****p* < 0.001; n = 3; Student’s two-tailed t test).

**Supplementary Figure. 5 FBXW2 regulates EGFR protein level.**

**a** Protein levels of EGFR and its downstream were markedly decreased or increased by FBXW2 overexpression or depletion in PCa cells. The DU145 and 22RV1 cells were transfected with targeted plasmids or siRNA, respectively, and followed by IB (ns, no significance, ***p* < 0.01; ****p* < 0.001; n = 3; Student’s two-tailed t test). **b** Neither FBXW2 overexpression nor depletion had any effect on the level of EGFR mRNA. The DU145 and 22RV1 cells were transfected with targeted plasmids or siRNA, respectively, and followed by qPCR (ns, no significance; n = 3; Student’s two-tailed t test). **c** Depletion of FBXW2 (si-FBXW2-#1014) extended protein half-life of endogenous EGFR in 22RV1 cells. After transfection with targeted siRNA for 48 h, 22RV1 cells were switched to fresh medium (10% FBS) containing cycloheximide (CHX) and incubated for indicated time periods before being harvested for IB. The band density was quantified using ImageJ software and plotted (****p* < 0.001; n=3; two-way ANOVA). **d** The levels of endogenously expressed EGFR and its downstream were reduced upon FBXW2 transfection, in a dose-dependent manner. The PC3 and DU145 cells were transfected with vector or plasmids with increasing amounts HA-FBXW2, and followed by IB. GAPDH levels served as the control for equal loading (ns, no significance; **p* < 0.05; ***p* < 0.01; ****p* < 0.001; n = 3; Student’s two-tailed t test).

**Supplementary Figure. 6 FBXW2 controls EGFR and its downstream pathways to suppress tumor growth *in vivo*.**

**a** IHC staining of xenograft tumor tissues. Tumour tissues from two groups of mice were fixed, sectioned and stained with indicated antibodies (**p* < 0.05; ***p* < 0.01; ****p* < 0.001; n = 3; Student’s two-tailed t test). Scale bars: 50μm. **b** Protein levels of EGFR and its downstream in of xenograft tumour tissues. Tumor tissues from two groups of mice were extracted protein and followed by IB. GAPDH levels served as the control for equal loading (ns, no significance; **p* < 0.05; ***p* < 0.01; ****p* < 0.001; n = 3; Student’s two-tailed t test).

**Supplementary Figure. 7 Overexpression of FBXW2 significantly abrogates invasion ability and proliferation caused by EGF.**

**a** Overexpression of FBXW2 significantly inhibited invasion ability caused by EGF. Transfected the vector control or plasmid expressing HA-FBXW2 for 48h in PC3 cells were treated with or without EGF. The invasion ability was detected by transwell assays (ns, no significance; ****p*<0.001; n = 3; one-way ANOVA). Scale bar: 100µm. **b** Overexpression of FBXW2 significantly inhibited cell proliferation caused by EGF (10ng/ml). Transfected the vector control or plasmid expressing HA-FBXW2 for 48h in PC3 cells were treated with or without EGF (10ng/ml). The cell ability was detected by CCK-8 assay (**p*<0.1, ****p*<0.001; n = 3; two-way ANOVA).

**Supplementary Figure. 8 FBXW2 blocks EGF-induced biological effects via its consensus degron motif.**

**a** Under EGF stimulation, transfected FBXW2 alone or co-transfection with wild-type EGFR suppressed invasion ability in DU145 cells, but was abrogated by simultaneous transfection of EGFR-MU2. Transfected HA-FBXW2 alone or in combinations with FLAG-EGFR (WT versus MU2) into DU145 cells and then treated with EGF (10ng/ml). The cell invasion ability was detected by transwell assays (ns, no significance; ****p*<0.001; n = 3; one-way ANOVA.), respectively. Scale bar: 100µm. **b** Under EGF stimulation, transfected FBXW2 alone or co-transfection with wild-type EGFR suppressed proliferation in DU145 cells, but was abrogated by simultaneous transfection of EGFR-MU2. Transfected HA-FBXW2 alone or in combinations with FLAG-EGFR (WT versus MU2) into DU145 cells and then treated with EGF (10ng/ml). The cell proliferation ability was detected by CCK-8 assays (****p*<0.001; n = 3; two-way ANOVA).
