# Supplementary figures and images for: FBXW2 inhibits prostate cancer proliferation and metastasis via promoting EGFR ubiquitylation and degradation
Source: Cell Mol Life Sci. 2022 May 2;79(5):268. doi: 10.1007/s00018-022-04320-3 (PMC9061686; doi:10.1007/s00018-022-04320-3)

**Figure S1**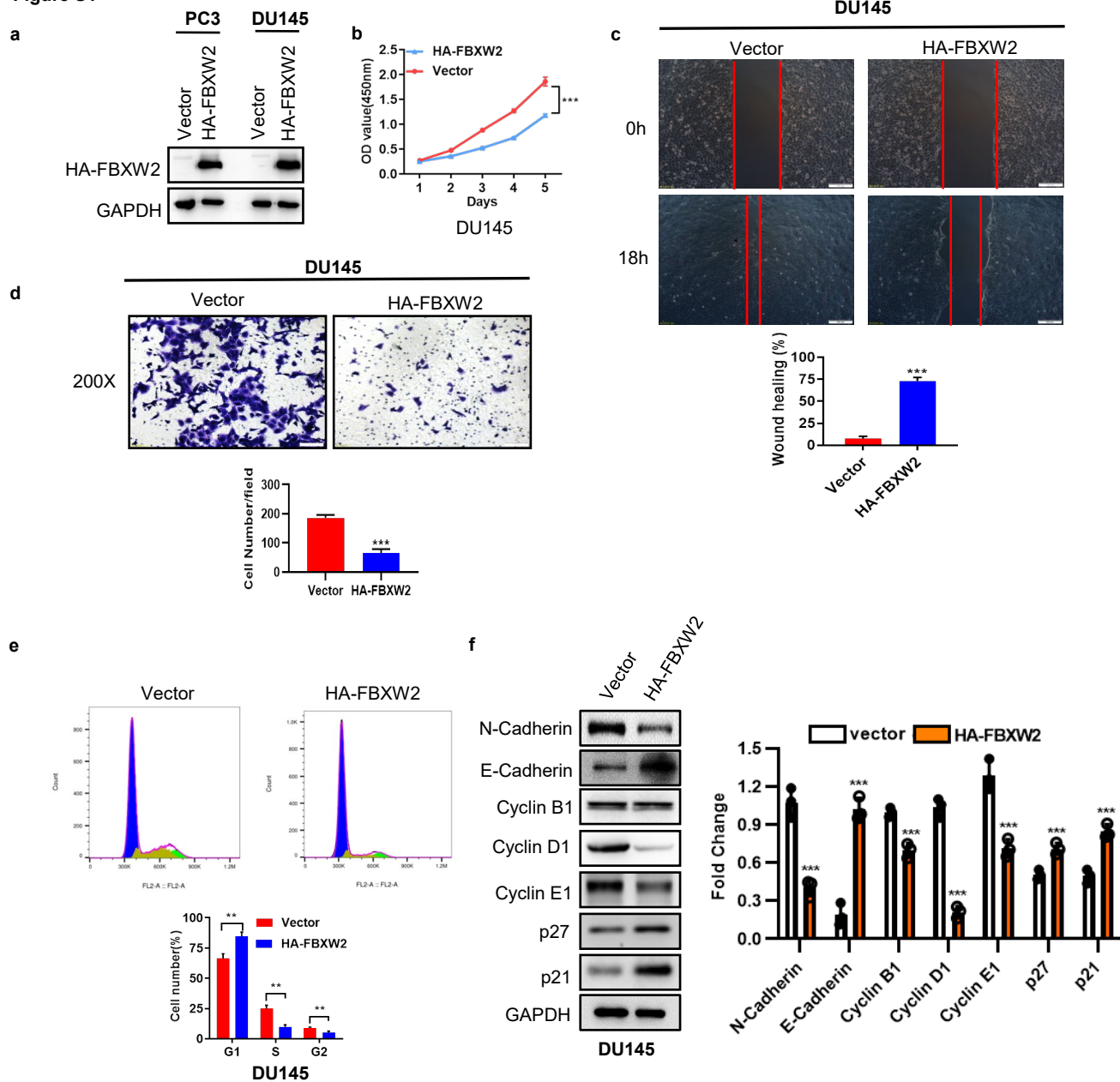

**Figure S2**

**a**

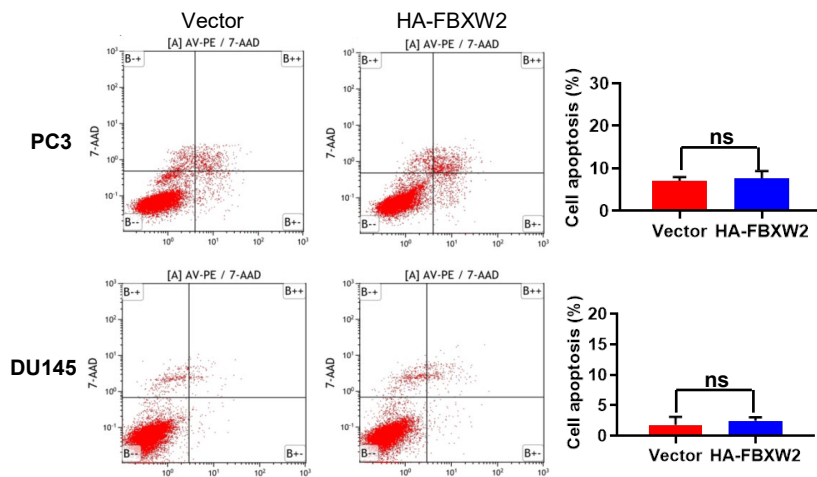

**b**

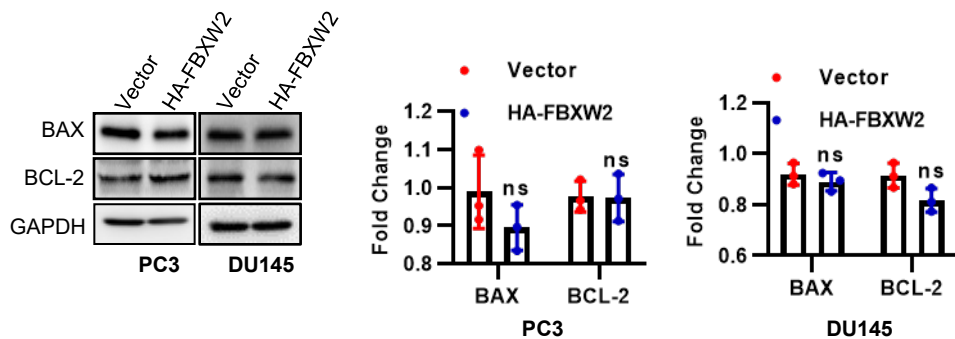

Figure S3

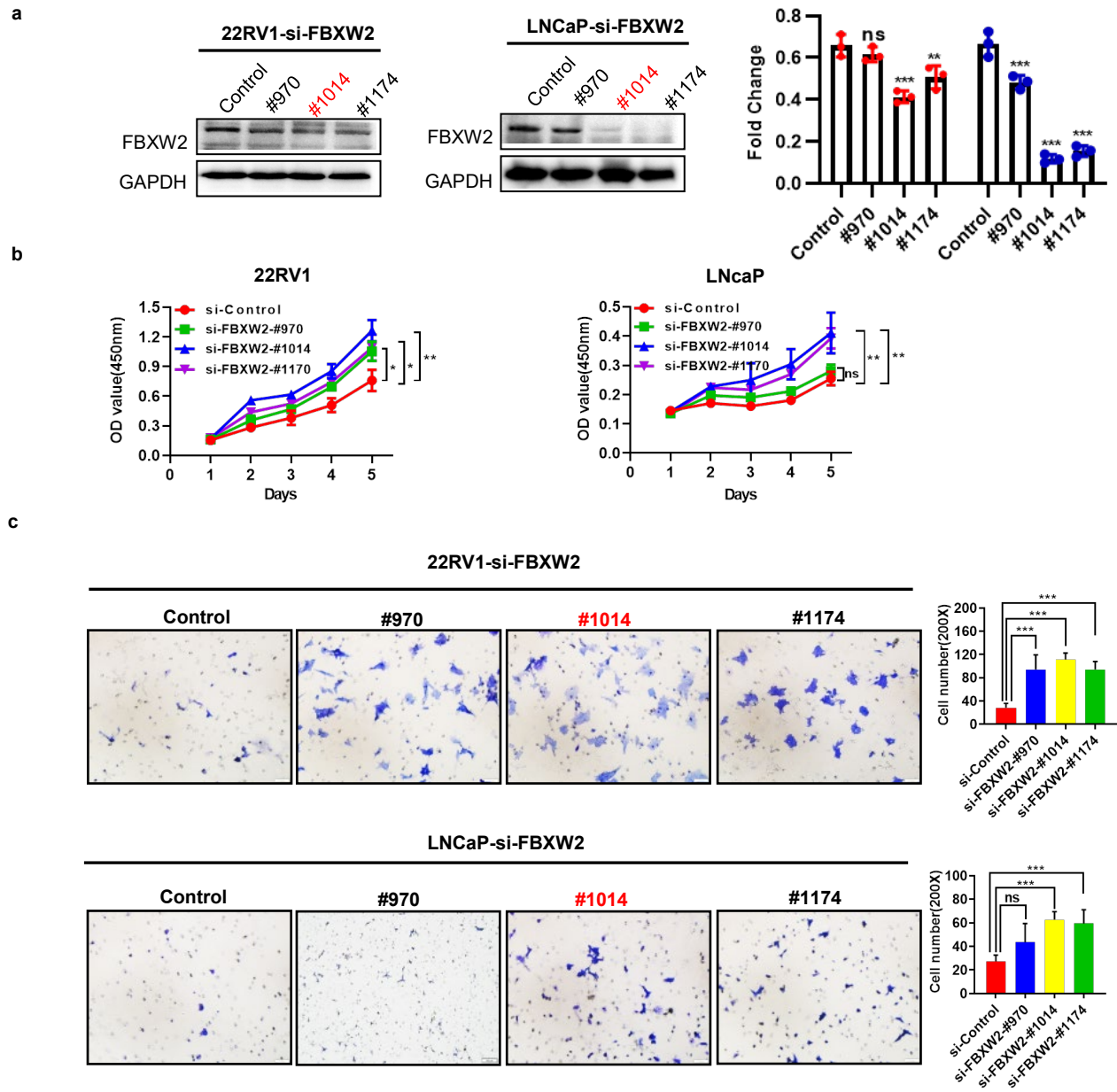

**Figure S4**

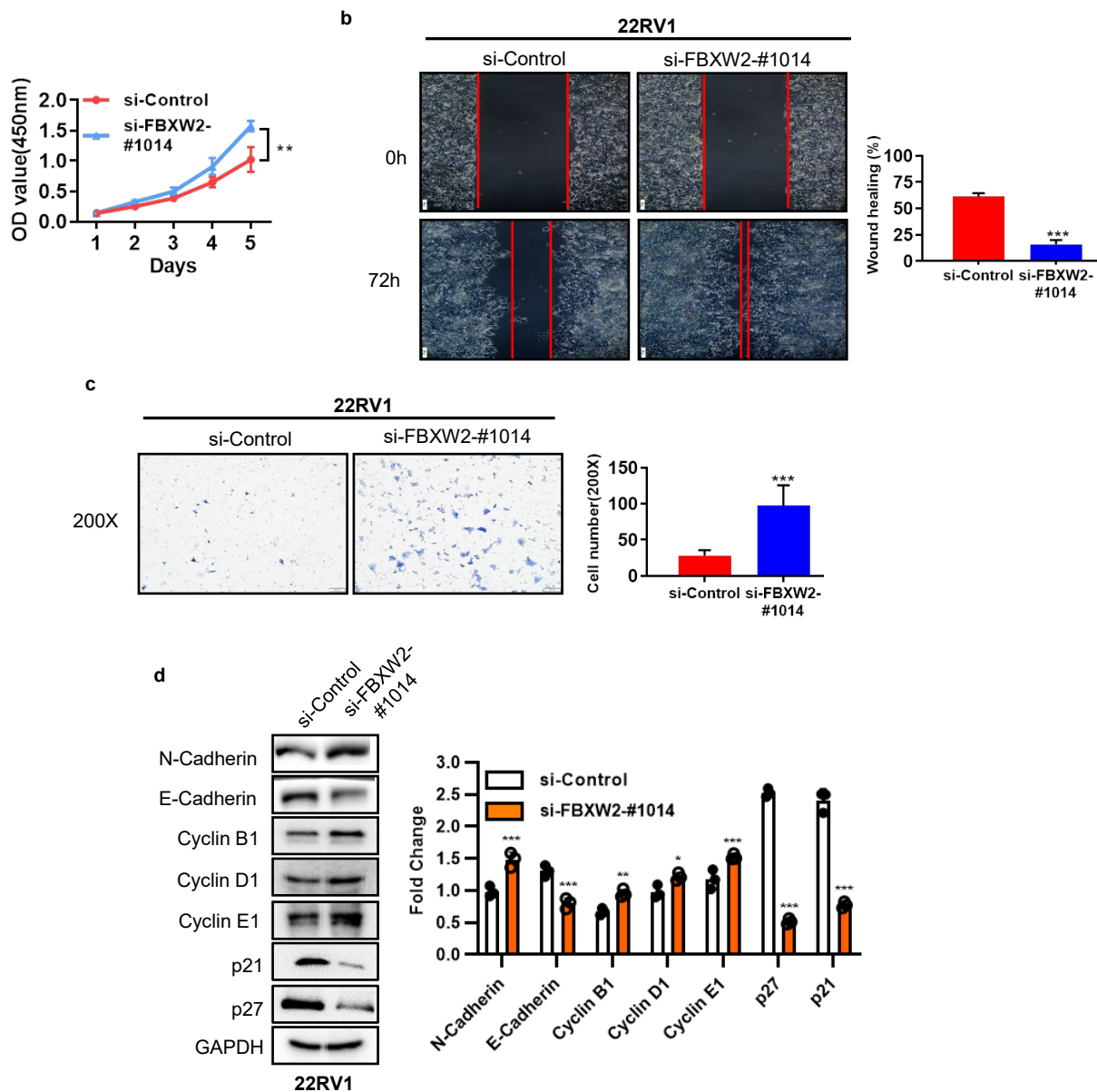

**Figure S5****a**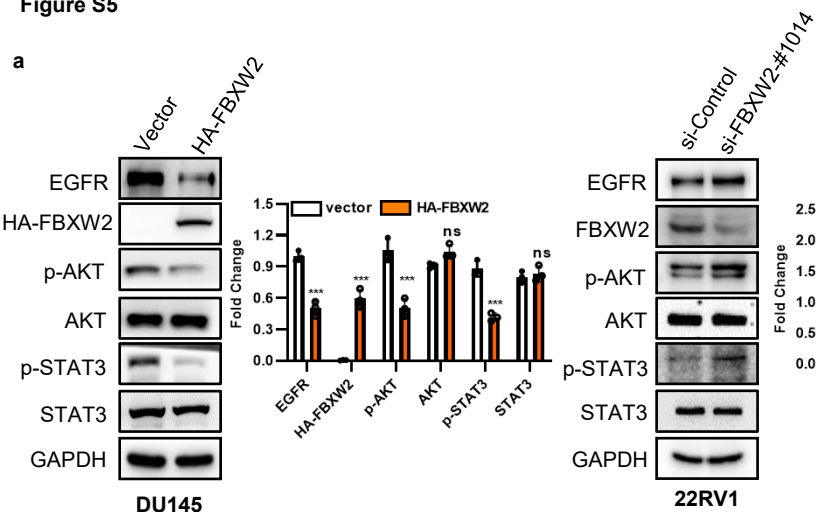**b**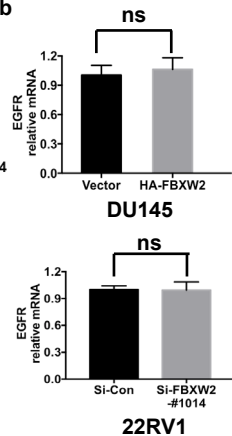**c**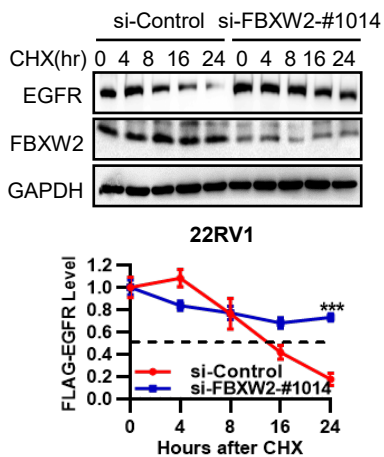**d**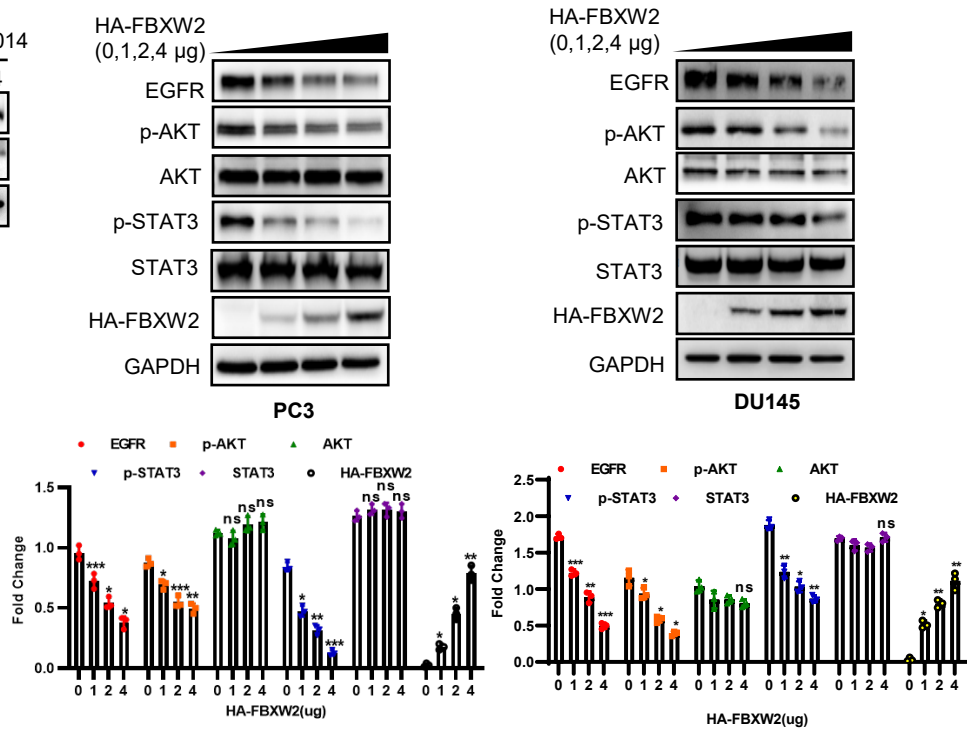

**Figure S6**

**a**

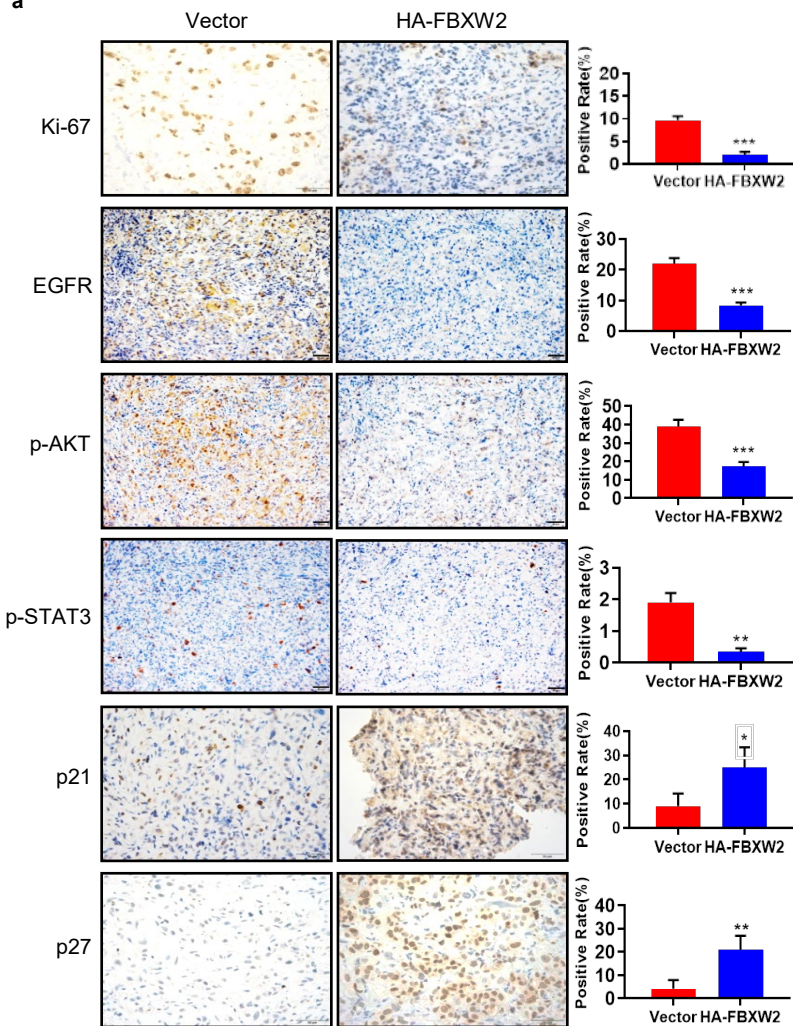

**b**

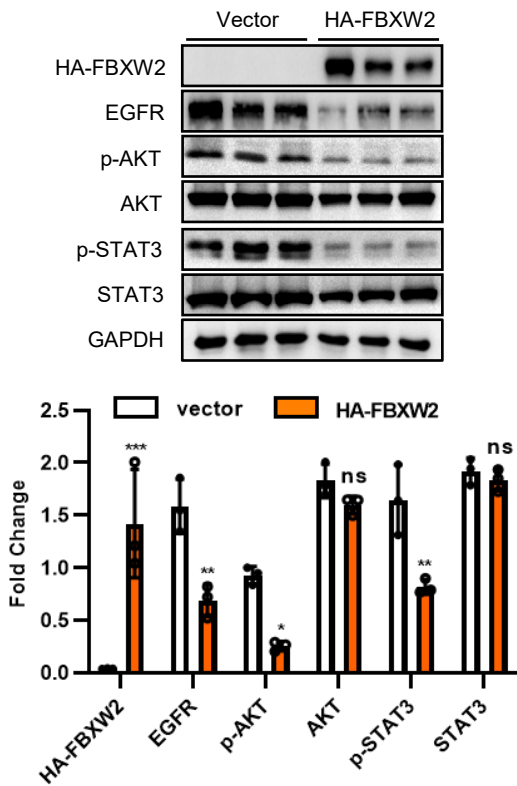

Figure S7

a

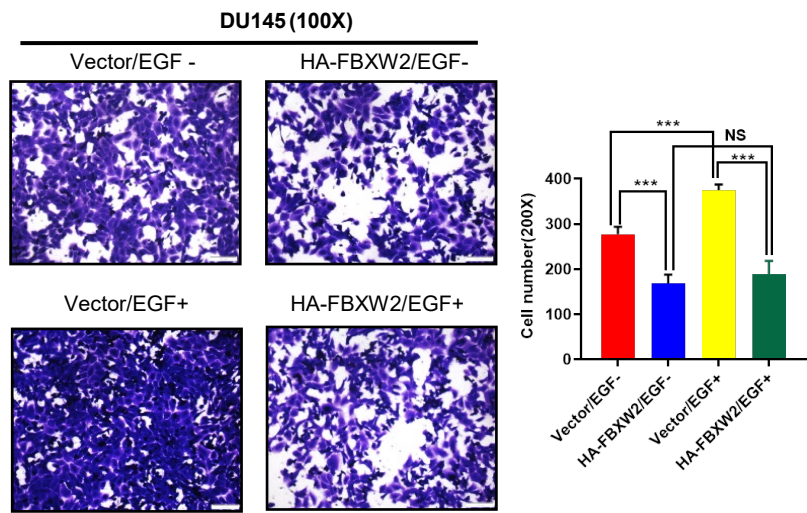

b

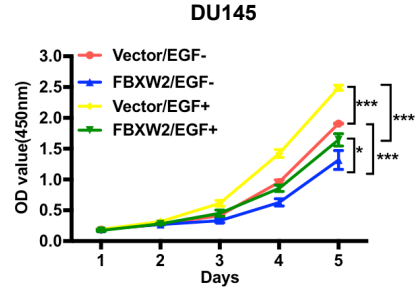

Figure S8

a

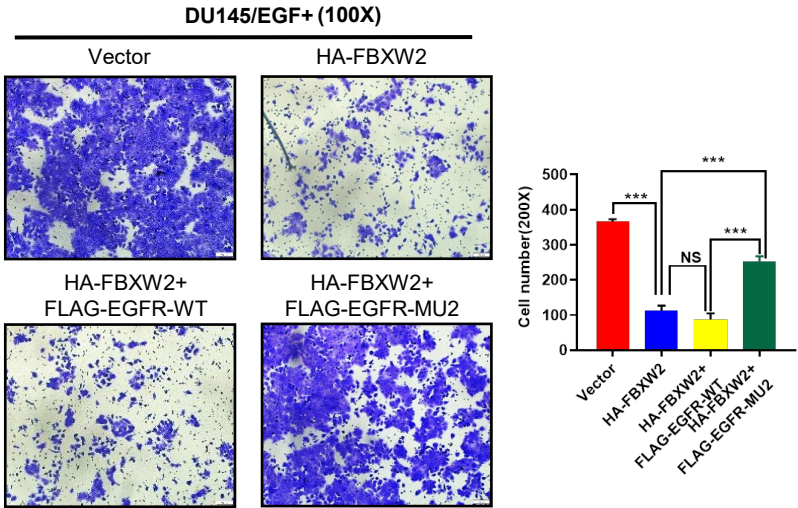

b

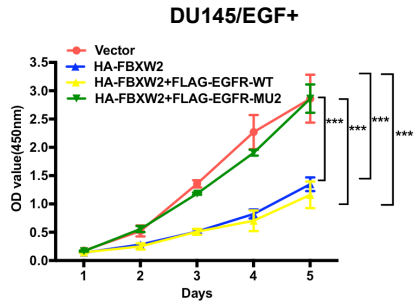

Supplement: Supplementary file 2 — Supplementary file2 (PDF 1838 KB) [file 18_2022_4320_MOESM2_ESM.pdf]

Figure1

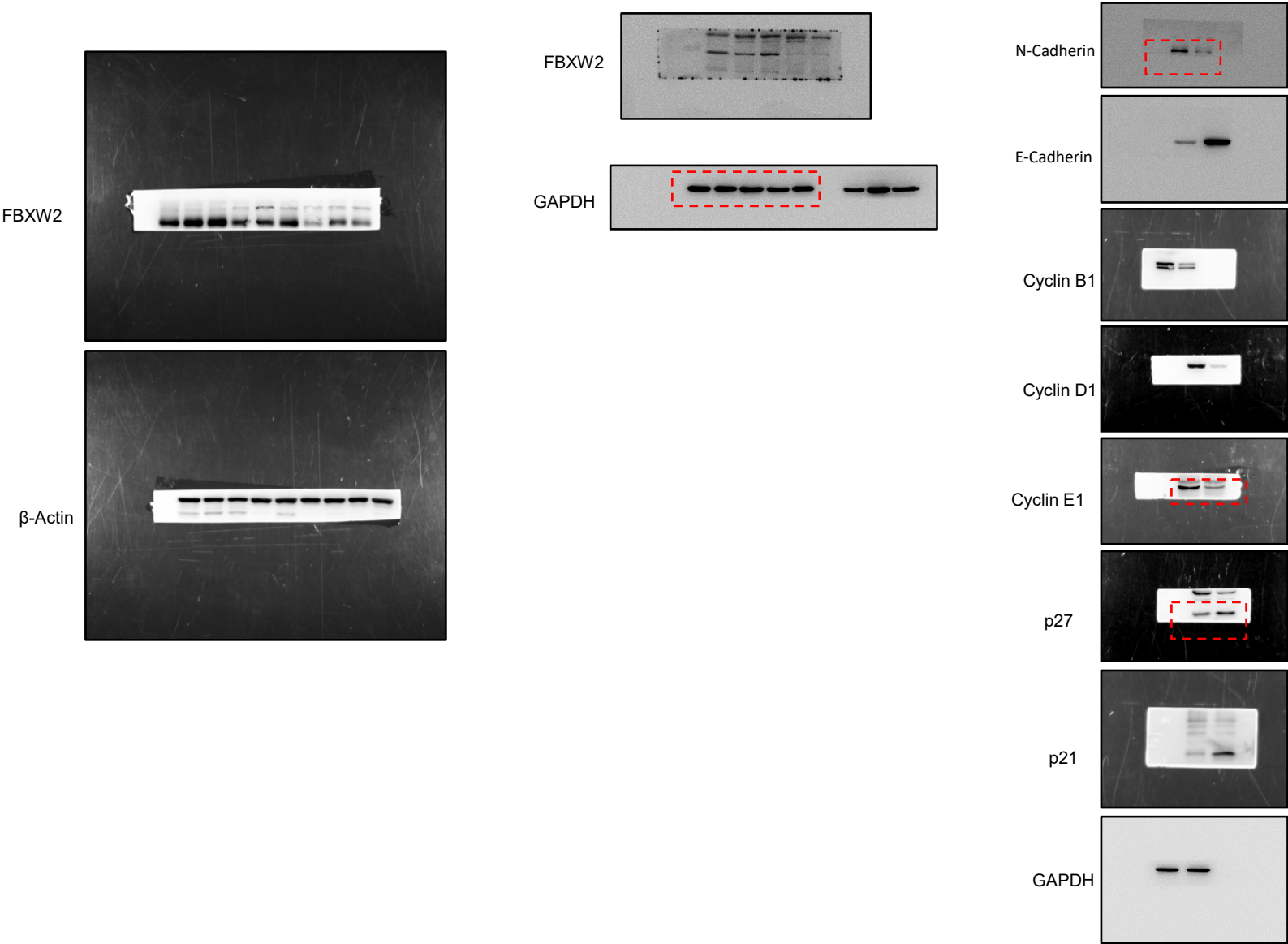

Figure3

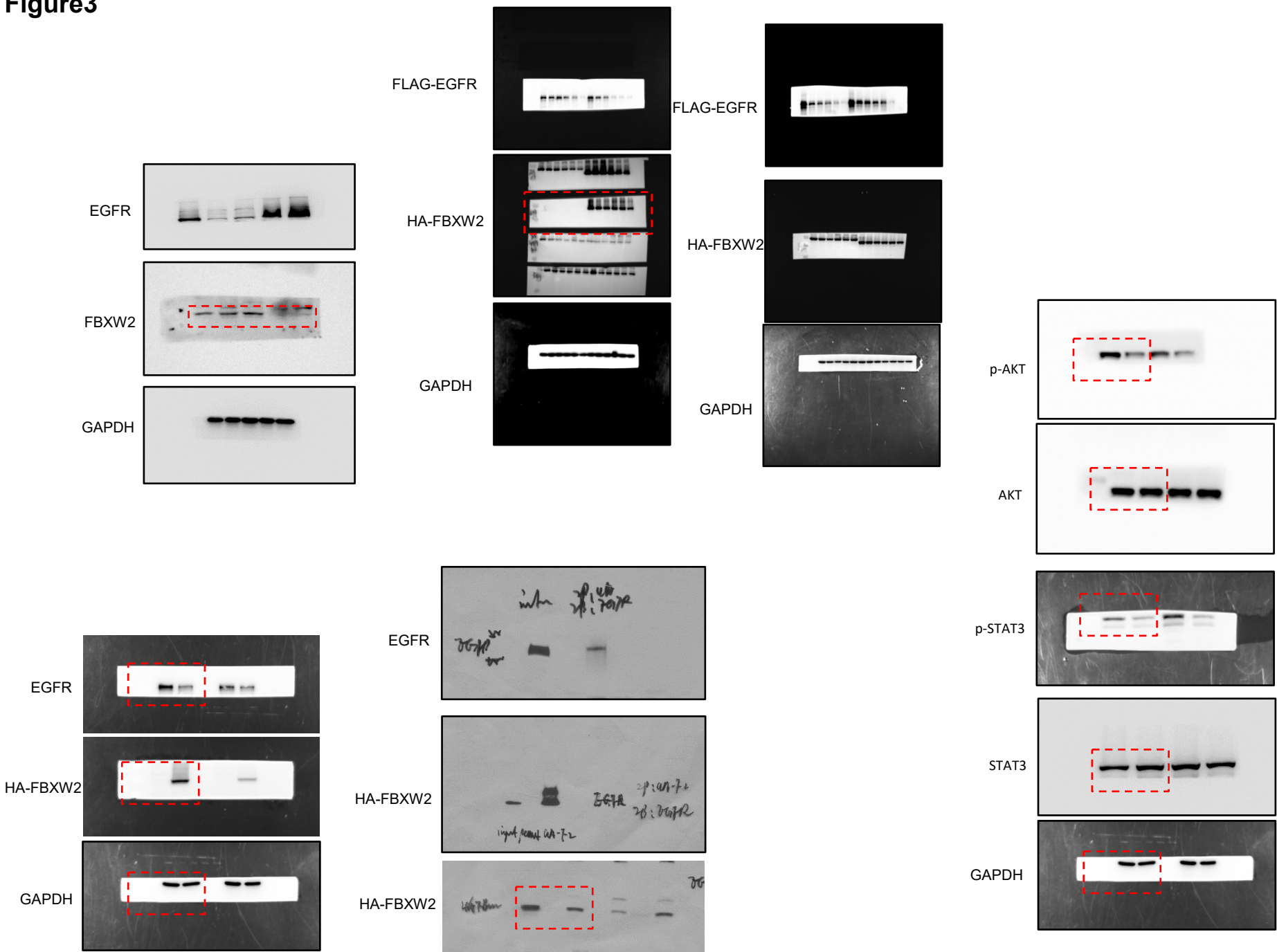

Figure4

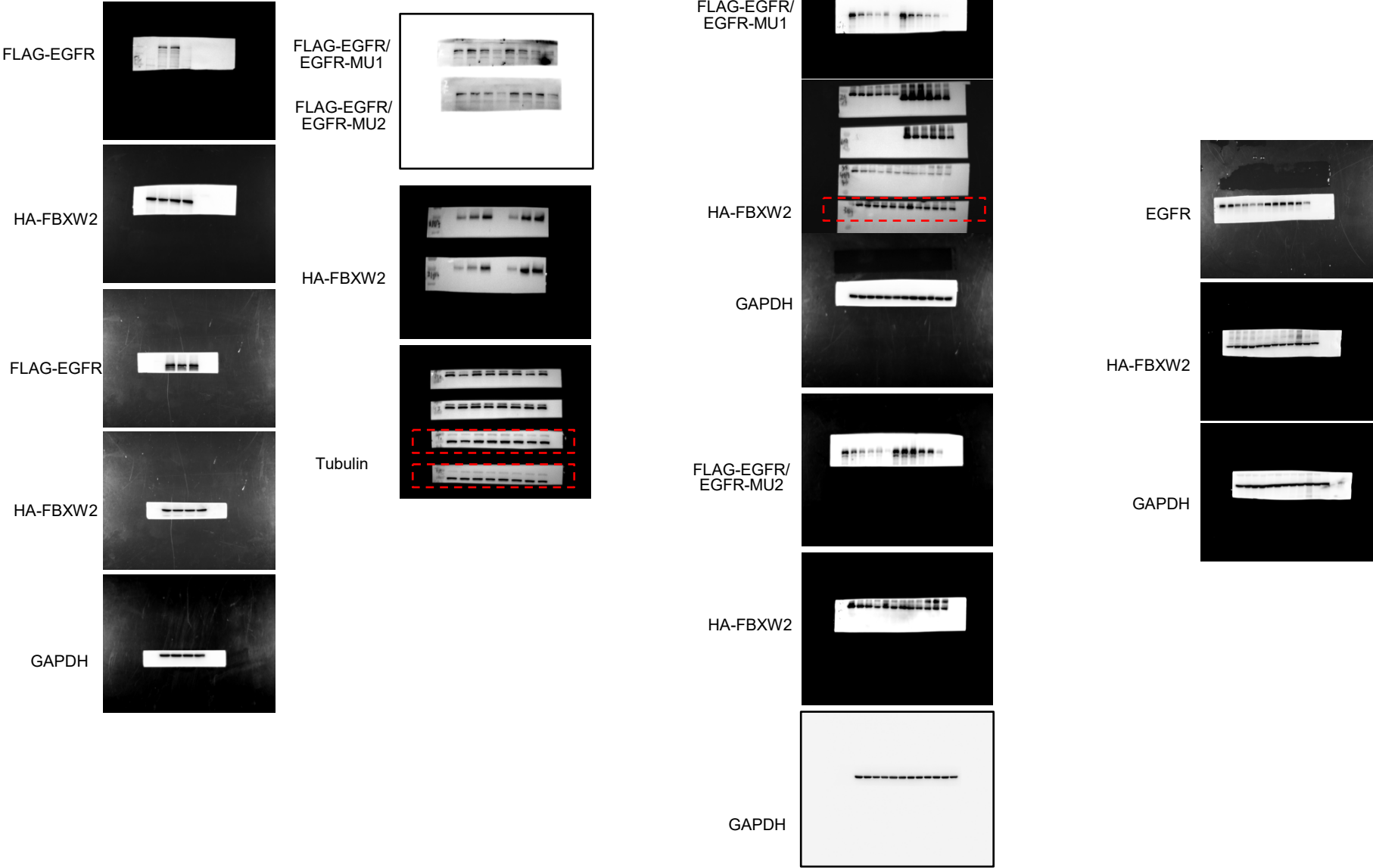

Figure5

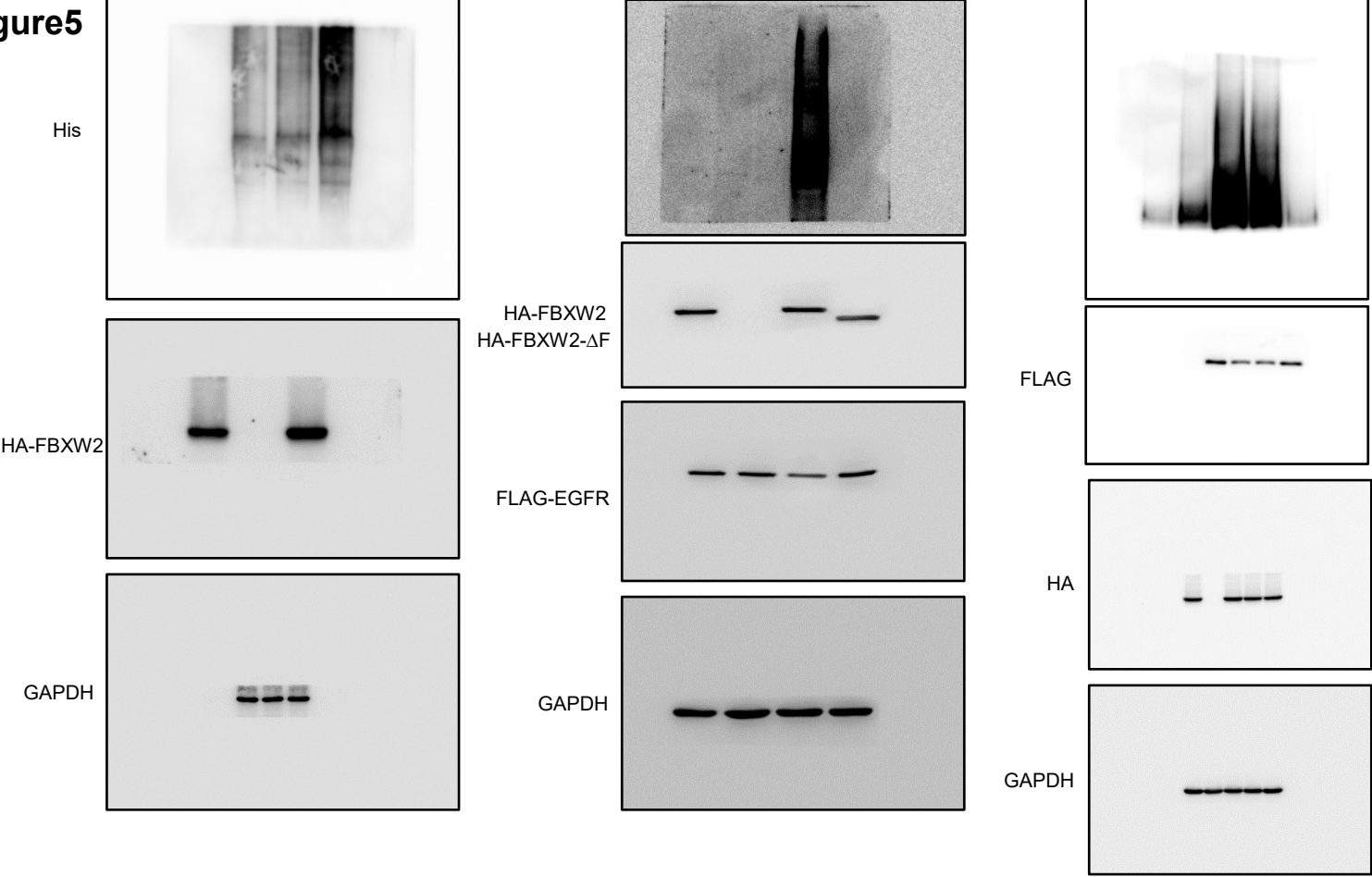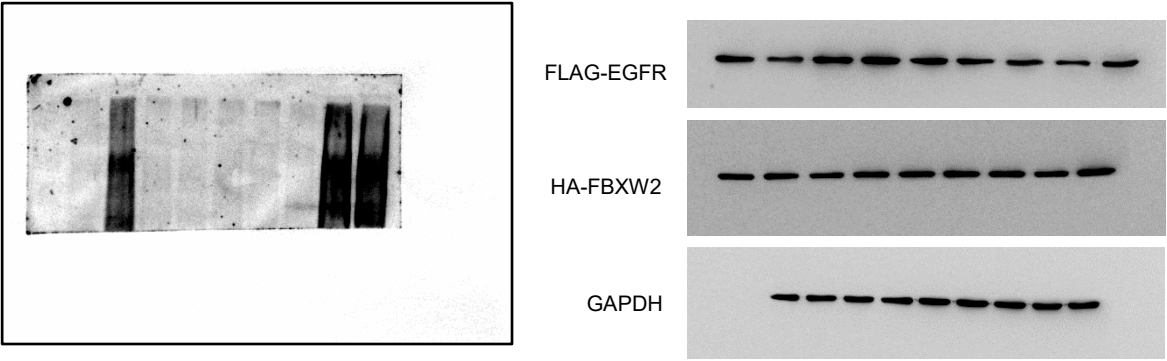

FigureS1

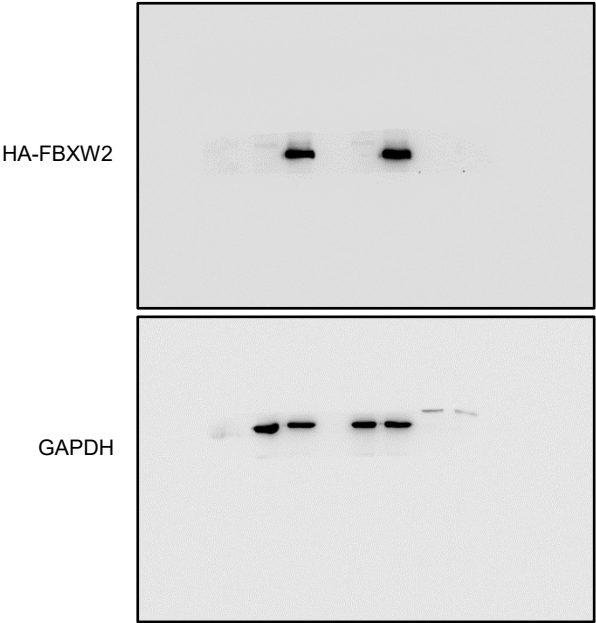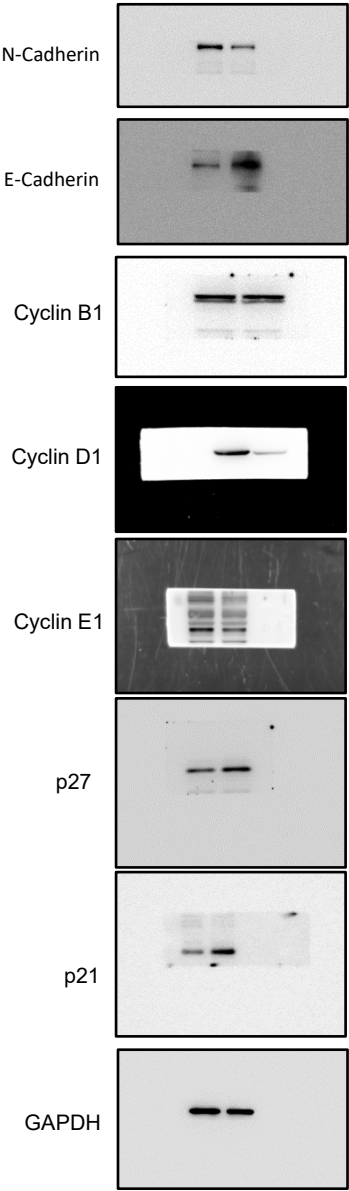

FigureS2

BAX

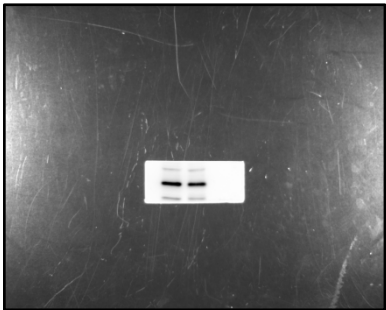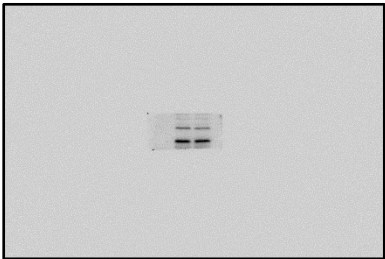

BCL-2

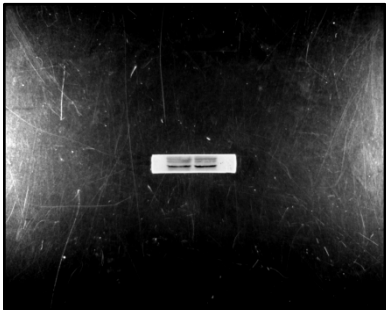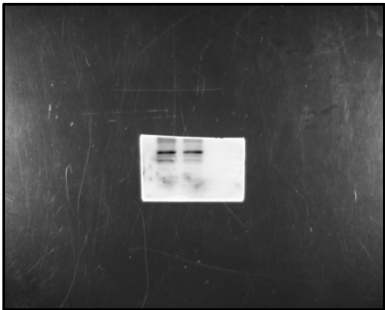

GAPDH

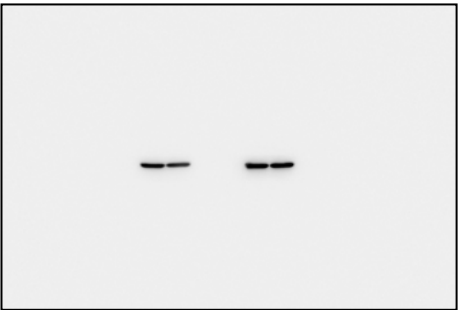

FigureS3

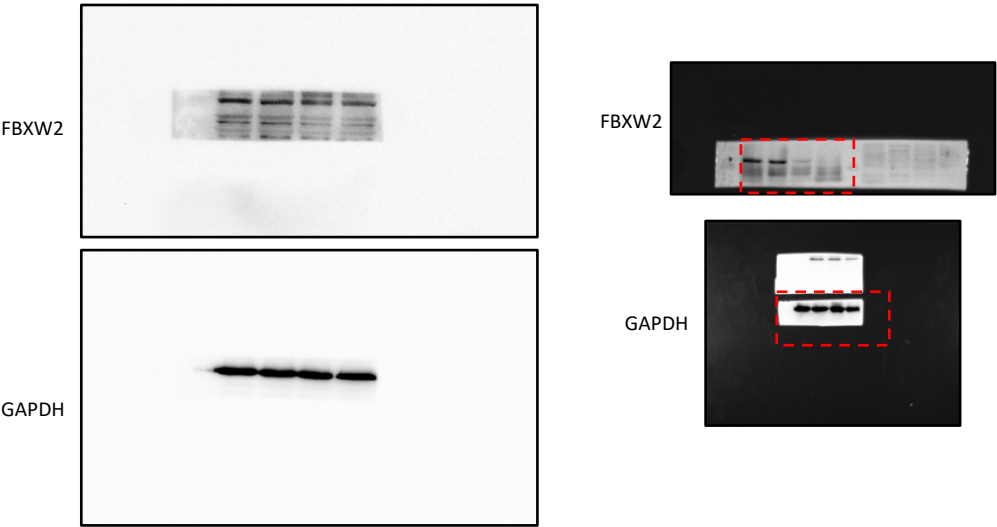

FigureS4

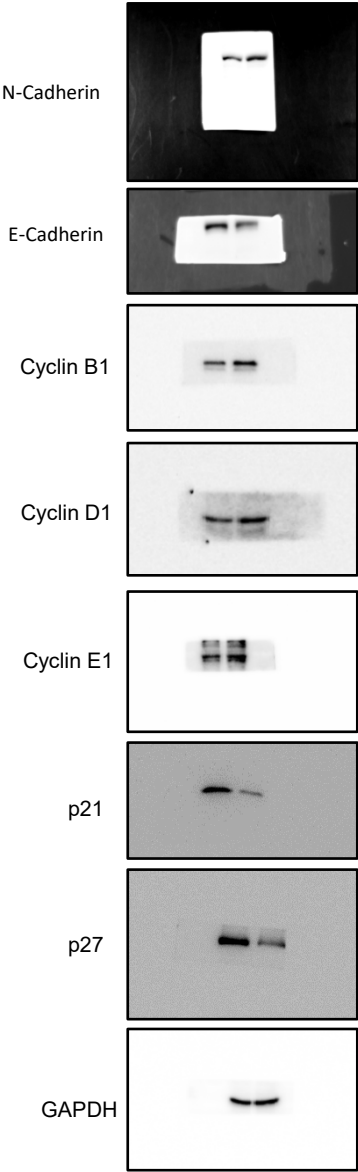

FigureS5

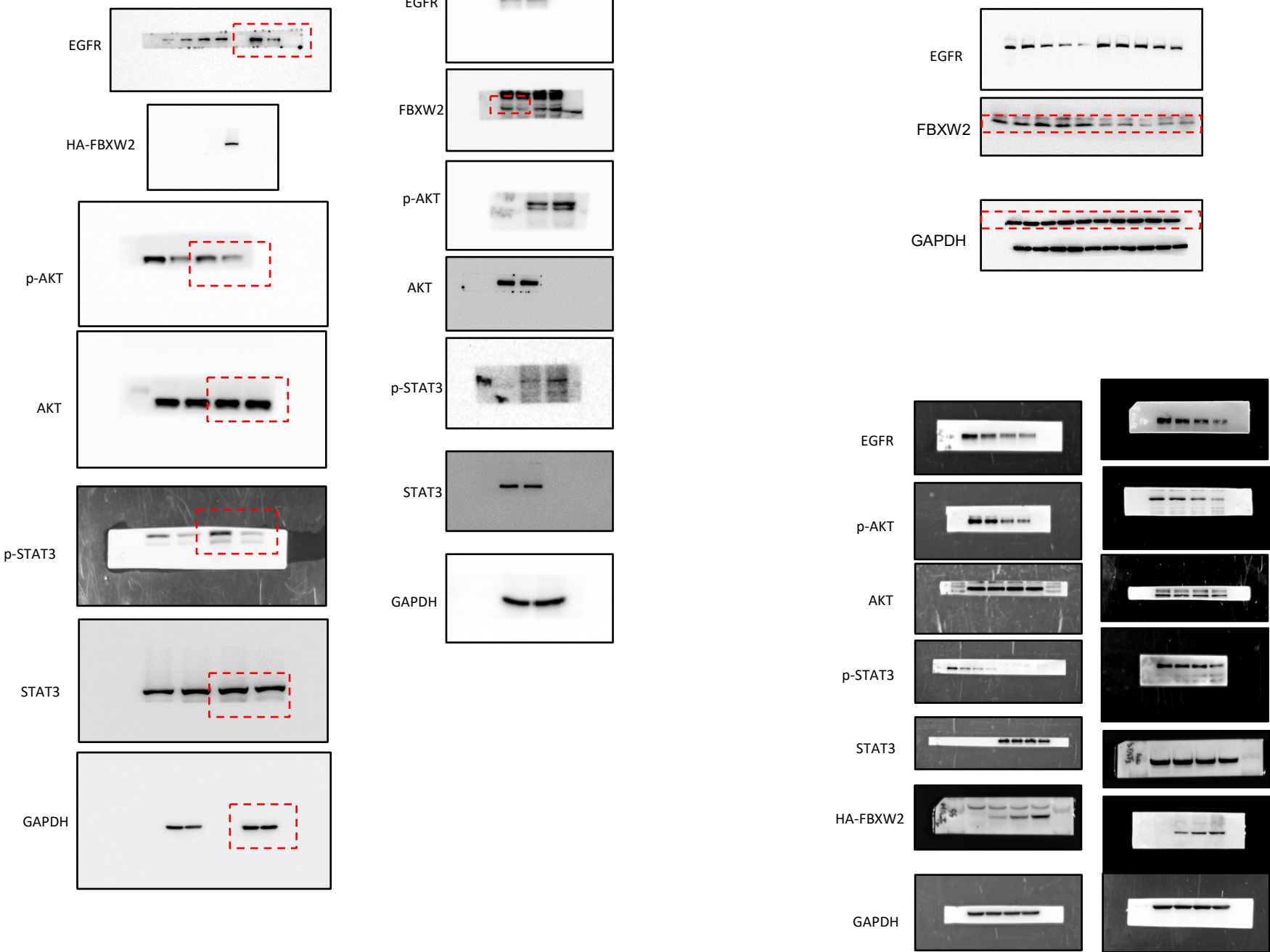

FigureS7

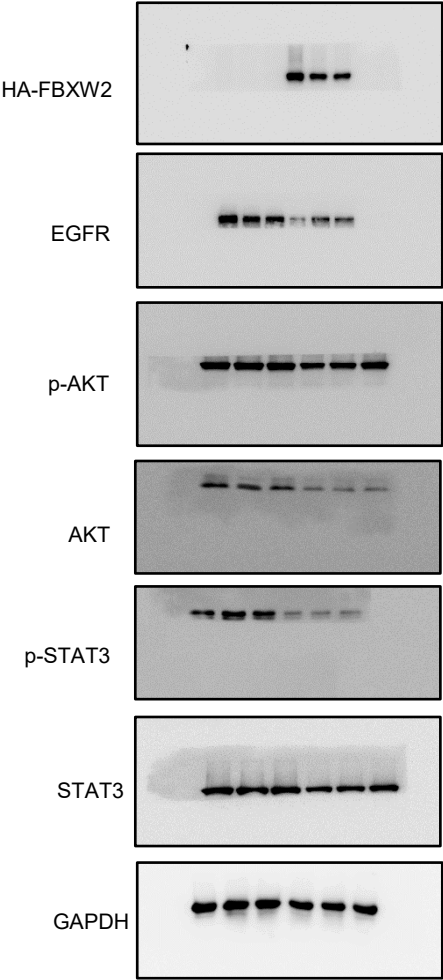

Supplement: Supplementary file 3 — Supplementary file3 (PDF 2553 KB) [file 18_2022_4320_MOESM3_ESM.pdf]
